# Substituent Effects Govern the Efficiency of Isoxazole Photoisomerization to Carbonyl‑2*H*‑Azirines

**DOI:** 10.1021/acsorginorgau.5c00105

**Published:** 2025-12-01

**Authors:** Kyra E. Jackson, Isabelle Szeto, Leah M. Seebald, Samuel G. Shepard

**Affiliations:** Department of Chemistry, 3776 Haverford College, 370 W Lancaster Ave, Haverford, Pennsylvania 19041, United States

**Keywords:** Photoisomerization, isoxazole, 2*H*-azirine, oxazole, atom-economy

## Abstract

The photoisomerization of isoxazoles is an atom-economical route to carbonyl-2*H*-azirines, which are valuable in both synthetic and biological applications. However, isolation of the carbonyl-2*H*-azirine is challenged by reverse photoisomerization back to the isoxazole and irreversible rearrangement to an oxazole. In this work, we demonstrate that substituent selection on 3,5-disubstituted isoxazoles plays a critical role in driving the photochemical isoxazole–azirine equilibrium toward the carbonyl-2*H*-azirine while avoiding oxazole formation. We find that substituents affect the degree of overlap in the absorption spectra of isoxazole–azirine pairs, where reducing overlap increases the efficiency of photoisomerization. We use time-dependent density functional theory to predict absorption spectra for isomer pairs with varied 3,5-disubstituents, identifying *tert*-butyl- and trifluoromethyl-substituted 5-aminoisoxazoles as promising structures. We then tested these predictions experimentally, revealing efficient formation of carbonyl-2*H*-azirines in high yields with minimal oxazole formation. This is in contrast to a phenyl-substituted 5-aminoisoxazole, which was found to readily form oxazoles, precluding isolation of the carbonyl-2*H*-azirine. These results demonstrate the utility of substituent-driven design for tuning photoisomerization equilibria and provide an atom-economical option for generating carbonyl-2*H*-azirines on synthetically useful scales.

## Graphical abstract

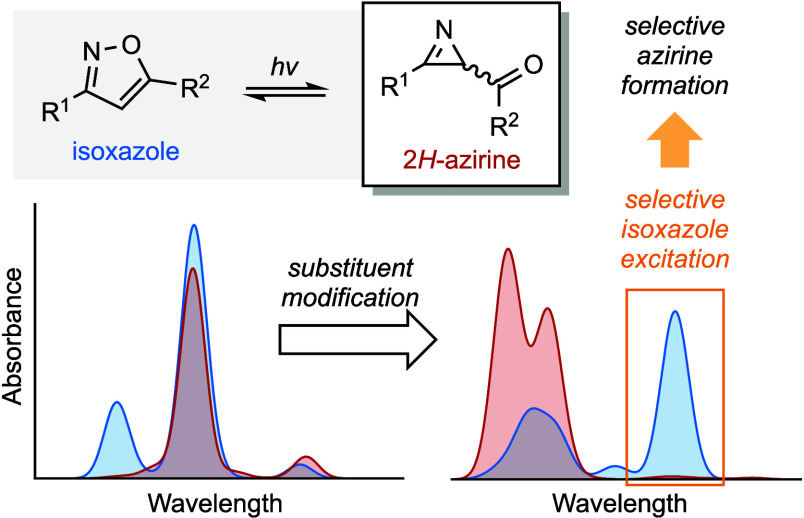

N-heterocycles are fundamental tools in pharmaceutical development, often serving as privileged scaffolds in bioactive agents. Expanding the accessible chemical space of N-heterocycles is critical for designing new molecules of pharmaceutical and broader interest; this expansion can be achieved by developing new synthetic methods to efficiently access more complex targets. For example, the 2*H*-azirine is a highly strained ring that serves as a versatile tool in the synthesis of a wide array of N-heterocycles.^1,2^ In addition to synthetic utility, 2*H*-azirines have also shown promise in biological applications, such as activity-based protein profiling (ABPP).^3,4^ Within this context, carbonyl-2*H*-azirines are of particular interest, as these moieties are found in a small set of natural products^3,4^ invoked for antimicrobial development.^2^ Given these applications, methods to safely and efficiently access carbonyl-2*H*-azirines are valuable.

Although there are several reported methods to synthesize carbonyl-2*H*-azirines,^5–9^ a green method to access this moiety is the photoisomerization of isoxazoles (Scheme 1).^10,11^ This photolysis results in cleavage of the isoxazole N–O bond to form a vinylnitrene intermediate, and an intramolecular biradical recombination yields the carbonyl-2*H*-azirine.^12–17^ This conversion demonstrates two of the 12 principles of green chemistry: the reaction has 100% atom economy and minimizes hazards, as the only added “reagent” is light.^18^ Despite these advantages, there are two drawbacks to synthesizing carbonyl-2*H*-azirines via photoisomerization. First, the isomerization from isoxazole to carbonyl-2*H*-azirine is photochemically reversible, where returning to the aromatic isoxazole can relieve the ring strain of the 2*H*-azirine moiety. Second, the carbonyl-2*H*-azirine can undergo an irreversible photoisomerization to an oxazole isomer. As a result, yields of carbonyl-2*H*-azirines are often low, with undesired rearrangements to oxazoles or ring-opening side products.^19–23^

**Scheme 1:**
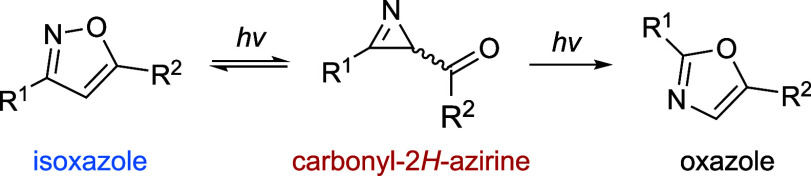
Photochemical Relationship between the Isoxazole, Carbonyl-2*H*-Azirine, and Oxazole Isomers

Since the seminal paper demonstrating the formation of carbonyl-2*H*-azirines via photoisomerization of isoxazoles,^24^ several authors have encountered the relationship between isoxazoles and 2*H*-azirines with little control over equilibria^19,25^ or worked to direct the pathway away from oxazoles using metal catalysts.^6,26–28^ In the original demonstration of this method, Singh and Ullman investigated the photochemical transformation of 3,5-diphenylisoxazole. The authors identified a carbonyl-2*H*-azirine intermediate in the transformation from isoxazole to oxazole. Illumination with a 254 nm light source excited 3,5-diphenylisoxazole, triggering isomerization to the corresponding carbonyl-2*H*-azirine. Subsequently, the same 254 nm light source excited the carbonyl-2*H*-azirine, inducing rearrangement to the oxazole.^24^ These results suggest that the overlap in the absorption spectra of isoxazole–azirine pairs could be critical to directing product formation. We posit that this overlap is the principal factor complicating the isolation of carbonyl-2*H*-azirines in the photoisomerization of isoxazoles.

The overlap in absorption spectra is likely caused by the conjugation of aryl substituents to the heterocyclic cores of the isomers, which constitutes the overwhelming majority of experimental isoxazole–carbonyl-2*H*-azirine photoisomerization studies.^20^ Aryl substituents, broadly useful in chemistry for the stability and tunability they provide, are often employed in photochemistry to enhance chromophore absorption due to “antenna effects.” ^29,30^ In this instance, however, the aryl substituents dominate the photochemical behavior of the isoxazole and the intermediate carbonyl-2*H*-azirine, leading to significant overlap of the absorption bands. Computational tools such as time-dependent density functional theory (TD-DFT) provide a facile method for predicting the effect of aryl substitution on the absorption spectra of isoxazole, carbonyl-2*H*-azirine, and oxazole.

To narrow our investigation, we focused on 3,5-disubstituted isoxazoles (where R^1^ and R^2^ identify the 3′ and 5′ positions in the isoxazole, respectively) and the corresponding carbonyl-2*H*-azirine and oxazole isomers (see back to Scheme 1). Specifically, we compared the computational absorbance predictions for isoxazole–azirine–oxazole isomer trios bearing aryl substituents against isomer trios bearing nonaryl groups (Figure S2). In our initial screen, amines in the R^2^ position were found to significantly enhance the discrimination between the isoxazole and carbonyl-2*H*-azirine absorbances when paired with alkyl groups in the R^1^ position. The R^2^-amino-isoxazoles could also easily be synthesized from α-cyanoketones and provide an alkylation handle for synthesizing more complex isoxazoles. This amino substituent provided a synthetically accessible starting point for validating our computational results. To more deeply analyze the effects of conjugation, we selected a set of isomer trios bearing primary amines in the R^2^ position with phenyl (Ph), *tert*-butyl (*t*-Bu), and trifluoromethyl (CF_3_) substituents in the R^1^ position as isomer trios **1**, **2**, and **3**, respectively. For reference of specific isomer trios, the following naming conventions will be used: Each isoxazole (**Is-1**, **Is-2**, **Is-3**) rearranges to the corresponding carbonyl-2*H*-azirine (**Az-1**, **Az-2**, **Az-3**, respectively) and oxazole (**Ox-1**, **Ox-2**, **Ox-3**, respectively). Predicted spectra for these isomer trios were generated using TD-DFT at a level of theory consistent with literature on heterocycles.^31^ The predictions revealed stark differences in the absorption spectra of isomer trio **1** compared with trios **2** and **3** (Figure 1). The lowest-energy major absorption band for the isoxazole, which guides the excitation wavelength for the photoisomerization experiment, exhibits a substantial bathochromic (red) shift for **Is-2** (222 nm) and **Is-3** (239 nm) relative to the corresponding carbonyl-2*H*-azirine, **Az-2** (179 nm) and **Az-3** (175 nm). This results in 40–60 nm separation between relevant isoxazole and carbonyl-2*H*-azirine absorption bands for **2** and **3**, providing a window to selectively excite the isoxazoles. This is in contrast to isomer trio **1**, for which there are no unique regions where **Is-1** absorbs but **Az-1** does not. These simulations predict that **Is-2** and **Is-3** can be selectively excited to yield **Az-2** and **Az-3** without excitation of the carbonyl-2*H*-azirine. Conversely, excitation of **Is-1** is predicted to occur at the same wavelength as excitation of **Az-1**, theoretically resulting in a mixture of **Az-1** and **Ox-1**. While the oxazole absorption bands are not relevant for isoxazole-to-azirine isomerization, it is worth noting that the calculated oxazole absorption bands for all three isomer trios show a bathochromic shift relative to those of the isoxazole and carbonyl-2*H*-azirine. This distinct absorbance is a useful handle for monitoring any oxazole formation in the isoxazole-to-azirine pathway.

**Figure 1:**
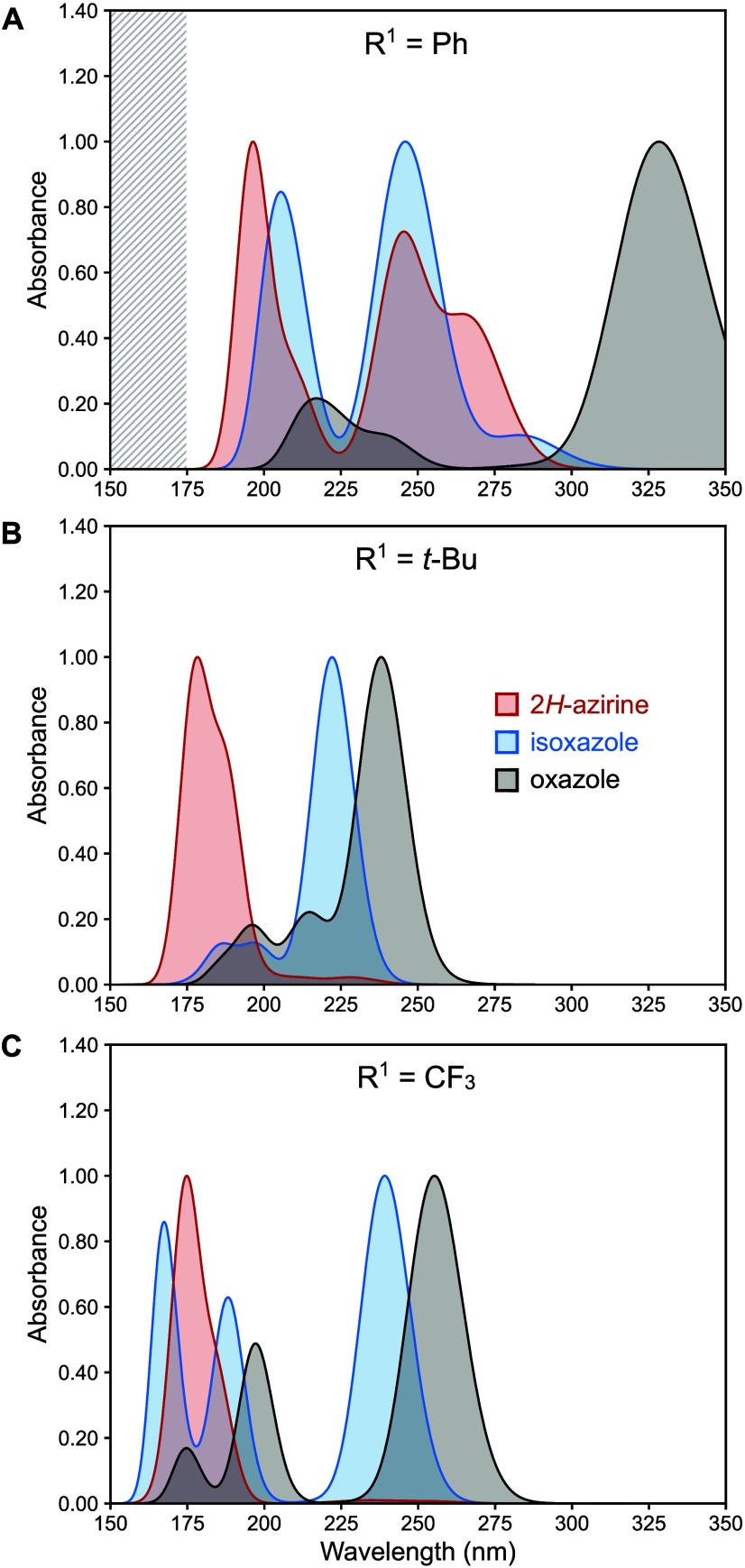
Predicted absorption spectra for isomer trios **1**, **2**, and **3** calculated at the TD-B3LYP/6–311+G­(2d,p)+PCM­(MeCN)// B3LYP/6–31G­(d) level of theory (see SI for additional details). (A) Spectra for **1**, the Ph-substituted isomer trio. (B) Spectra for **2**, the *t*-Bu-substituted isomer trio. (C) Spectra for **3**, the CF_3_-substituted isomer trio. The gray bar in (A) indicates a region in which no additional roots were calculated by TD-DFT.

To experimentally validate these computational findings, we conducted a series of photoisomerization experiments on the three isomer trios, **1**–**3**, discussed above. The starting isoxazole compounds were either synthesized (**Is-1** and **Is-2**) or purchased from commercial suppliers (**Is-3**). A one-step cyclization of α-cyanoketones (3-oxo-3-phenyl-propionitrile and 4,4-dimethyl-3-oxo-pentanenitrile, respectively) with hydroxylamine hydrochloride gave **Is-1** and **Is-2** (Scheme 2).^32^ The computational and experimental spectra for the isoxazoles show good agreement (Figure S3), demonstrating the utility of TD-DFT for these absorbance predictions.

**Scheme 2:**
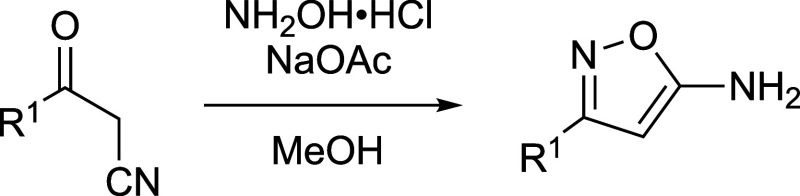
Synthetic Route Affording the Isoxazoles **Is-1** and **Is-2** from the Corresponding α-Cyanoketones

Isoxazoles **Is-1**, **Is-2**, and **Is-3** were subjected to UV photoisomerization with a 255 nm LED (see SI for details). The progress of each photoisomerization was monitored by UV–visible and ^1^H NMR spectroscopy (Figure 2). Full spectra, including extended photoisomerization times and ^19^F NMR for isomer set **3**, can be found in SI. Photoisomerization of **Is-1** shows the rapid growth of a new absorption band centered at 310 nm over the first 60 s of illumination (Figure 2A). Given the computational prediction of the **Ox-1** absorbance maximum at 329 nm, it seems feasible that this absorption peak detected by UV–vis is **Ox-1**. Prevailing computational work predicts that **Az-1** is an intermediate in the formation of **Ox-1**,^24,33,34^ but there is little evidence for this intermediate in the UV–vis data. This is likely due to similarities in absorption spectra for **Is-1** and **Az-1** (predicted λ_max_ of **Is-1** = 206, 246 nm, predicted λ_max_ of **Az-1** = 196, 246 nm).

**Figure 2:**
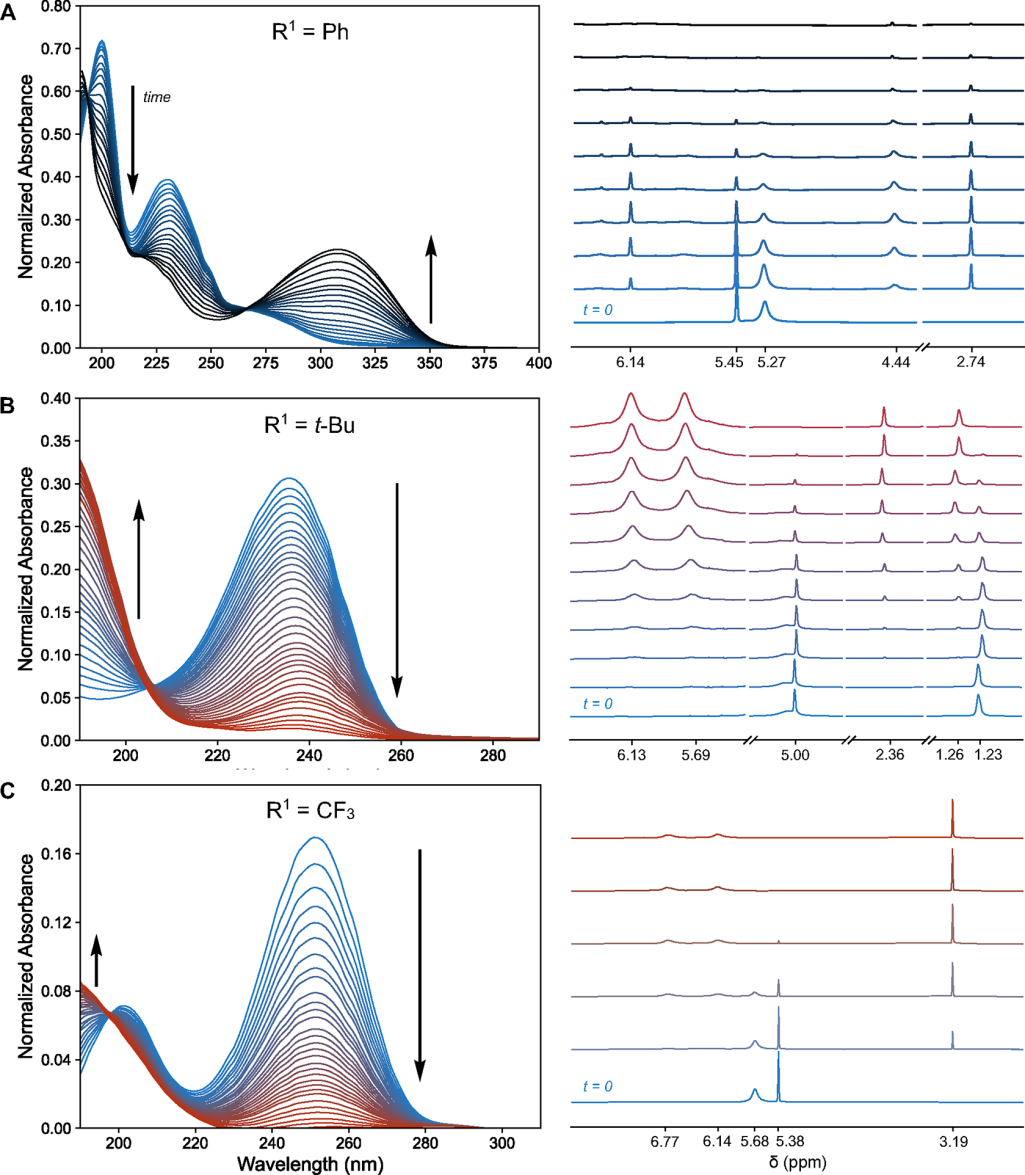
UV–vis and NMR photolysis progress for (A) **Is-1**, (B) **Is-2**, and (C) **Is-3**. For UV–vis scale experiments, samples were prepared at about 4 μg/mL in HPLC-grade MeCN. For NMR-scale experiments, samples were prepared at about 10 mg/mL in MeCN-*d*_3_. The stacked ^1^H NMR spectra are trimmed for clarity and hallmark regions are independently scaled for easier visualization. Hashed lines indicate trim points. For full spectra and additional photoisomerization details, see SI. (A) UV–vis data (left) show changes over 1 min and NMR data (right) show changes over 3 h. (B) UV–vis data (left) show changes over 15 min and NMR data (right) show changes over 8 h. (C) UV–vis data (left) show changes over 1 min and NMR data (right) show changes over 30 min.

To elucidate the pathway of **Ox-1** formation, we turned to ^1^H NMR monitoring of the reaction (Figure 2A). At the higher concentrations necessary for ^1^H NMR monitoring (10 mg/mL in MeCN-*d*_3_), the photoisomerization of **Is-1** occurs more slowly than at concentrations required for UV–vis (4 μg/mL in MeCN). The ^1^H NMR time course shows consumption of **Is-1** peaks (δ = 5.45, 5.27 ppm) by 20 min and the formation of new strong peaks at 6.14 and 4.44 ppm, integrating to a respective 1:2 ratio, consistent with a 5-amino-oxazole.^35–37^ The agreement of UV–vis and ^1^H NMR data gives us confidence that the rapidly formed species is **Ox-1.** At this time point, it is also notable that there are ^1^H NMR peaks associated with **Az-1** as distinguished by two broad peaks at 6.32 and 5.80 ppm, characteristic of the two amide protons, and the 2*H*-azirine proton at 2.74 ppm. Together, these experiments corroborate literature precedent that the carbonyl-2*H*-azirine is likely an intermediate in the photoisomerization of isomer trio **1**.^33,34,38^ These results suggest that isolation of a carbonyl-2*H*-azirine with a phenyl substituent at R^1^ is consistently challenged by the rapid rearrangement of the carbonyl-2*H*-azirine to the oxazole byproduct. In this way, the behavior of isomer trio **1** parallels 3,5-diphenylisoxazoles studied in prior work, where the principal route to the oxazole was predicted to require two photons and pass through an excited carbonyl-2*H*-azirine intermediate.^15,24^, Although isolation of **Ox-1** is beyond the scope of this work, we note that the NMR (Figure S6) and UV–vis spectra (Figure S7) continue to evolve beyond this early time scale due to suspected oxazole decomposition.

Turning next to the UV photoisomerization of **Is-2**, we observed a gradual decrease in the characteristic isoxazole absorbance centered at 235 nm over the course of 15 min, accompanied by the emergence of a new peak at about 190 nm, which is not fully resolved due to instrument limitations (Figure 2B). The slower rate of photoisomerization compared to **Is-1** may be due to an enhanced oscillator strength of the phenyl-conjugated species. It may also be due to a spectral mismatch of the λ_max_ of **Is-2** (235 nm) compared to the irradiation wavelength of the lamp (255 ± 5 nm) especially in light of the more rapid isomerization of **Is-3** (*vide infra*). In the photoisomerization spectra for **Is-2**, the presence of an isosbestic point suggests a one-to-one conversion from **Is-2** to a single product. This newly formed peak is not in the vicinity of the predicted **Ox-2** absorbance centered at 238 nm; instead, it resembles the predicted absorbance of **Az-2** at 179 nm. To support this identification, we monitored the reaction progress by ^1^H NMR (Figure 2B), which showed two diagnostic amide proton signals at 6.13 and 5.69 ppm that are consistent with **Az-2**, along with the characteristic 2*H*-azirine proton at 2.36 ppm (Figure S8). These data confirm that under these conditions **Az-2** is the major product of **Is-2** photolysis.

Finally, irradiation of **Is-3** with 255 nm light resulted in the complete consumption of the isoxazole absorbance centered at 251 nm and the formation of a peak in the deep UV range in less than 1 min (Figure 2C). Based on the calculated absorbance of **Az-3** (λ_max_ = 175 nm), the rise of absorbance below 200 nm, and the absence of any feature attributable to **Ox-3** (predicted λ_max_ = 255 nm), this species is consistent with **Az-3**. This assignment was further supported by both ^1^H NMR (Figure 2C, Figure S9) and ^19^F NMR (Figure S10). The isomer trios **2** and **3** exhibit comparable experimental photoisomerization properties, enabling the formation of carbonyl-2*H*-azirines with no detectable oxazole formation. This demonstrates that with appropriate substituent selection, carbonyl-2*H*-azirines can be cleanly accessed through a single-step photoisomerization of the corresponding isoxazole precursors, making this an exceptionally atom-efficient synthetic approach.

To demonstrate the synthetic viability of this substituent-guided photoisomerization approach, we scaled up the reaction of **Is-2** to 100 mg. **Az-2** was readily isolated as the major product after solvent removal without the need for chromatographic purification. This result highlights the scalability, efficiency, and simplicity of this method for accessing carbonyl-2*H*-azirines in synthetically useful quantities. We also scaled up the reaction of **Is-3** to 100 mg to obtain **Az-3.** Using the same procedure, **Az-3** was generated with no detectable oxazole byproduct. However, solvent removal resulted in apparent degradation, as evidenced by the significantly reduced isolated mass (<10% yield) and ^1^H and ^13^C NMR spectra that did not match the expected product. These findings suggest that **Az-3** is too reactive to be isolated under the conditions examined. Prior studies have shown that photochemical rearrangements of isoxazoles can generate highly reactive intermediates, including ketenimines,^39^ which can undergo rapid secondary reactions. Additionally, 2*H*-azirines are well-known to be reactive species capable of ring-opening, cycloaddition, or conversion to aziridines that may undergo polymerization or further ring opening reactions.^40,41^ The exact identity of the decomposition products observed here remains inconclusive based on available characterization data; however, the collective literature precedent indicates that multiple reactive pathways are plausible. Nonetheless, the described method reliably provides clean *in situ* generation of **Az-3** for immediate use.

Next, we sought to understand the electronic basis for the stark contrast between isomer trio **1** (Ph-substituted) and isomer trios **2** and **3** (*t*-Bu- and CF_3_-substituted). Focusing on just the isoxazole–azirine pairs, we analyzed the orbital character for primary transitions of interest to better understand trends in spectral overlap (Figure S4). Notably, the major absorption bands for isoxazoles **Is-2** and **Is-3** (primarily derived from a LUMO ← HOMO) feature an expansion of electron density from the isoxazole core and amino group to the entire molecule, including the R^1^ substituent, while the corresponding carbonyl-2*H*-azirine transitions show little change in electron density across the molecule. In contrast, for isomer pair **1**, both the isoxazole and carbonyl-2*H*-azirine major orbital transitions show a migration of electron density away from the amine. The similarities in orbital transitions for the isomer pair **1** result in overlapping absorbances around 245 nm. Effectively, both **Is-1** and **Az-1** behave like substituted benzene compounds, and thus differences in absorption spectra are minimal. As a result, the carbonyl-2*H*-azirine is eventually re-excited and goes on to engage in a second photoisomerization reaction.

The reversible photoisomerization of isoxazoles to carbonyl-2*H*-azirines and the further rearrangement to oxazoles has been well-documented, fueling interest in method development for efficient isolation of the carbonyl-2*H*-azirine species.^22,24,34^ Our work complements these approaches by identifying the key role of the absorption spectra of the isomer trios in determining the outcome of photolysis. When isomer trios are chosen with intentional substituent selection on the isoxazole, spectral separation enables selective photoisomerization of the carbonyl-2*H*-azirine. This separation prevents the re-excitation of the carbonyl-2*H*-azirine, avoiding the rearrangement to oxazoles that so often complicates the synthesis of aryl-substituted carbonyl-2*H*-azirines. To inform substituent selection, we have demonstrated the utility of TD-DFT as a predictive tool in the design of efficient synthetic routes to carbonyl-2*H*-azirines. Using this predictive tool, we have found that nonconjugated R^1^ groups and amino R^2^ groups are effective at engineering spectral separation such that the photoisomerization results in no detectable oxazole formation. We intend to expand this substituent investigation in further work to uncover the electronic dynamics driving this result, establishing concrete design principles for the photoisomerization of isoxazoles to carbonyl-2*H*-azirines. An electronically-guided synthetic design will ultimately enable atom-economical access to a broad range of structurally diverse carbonyl-2*H*-azirines.

## Supplementary Material



## Data Availability

The data underlying this study are available in the published article and its Supporting Information.

## Notes

Computational and synthetic methods, including characterization of new molecules (^1^H, ^13^C, and ^19^F NMR spectra), additional computational experiments, and extended reaction monitoring (PDF)

## †

Department of Chemistry, University of Chicago, Chicago, Illinois 60637, United States

## ‡

Department of Chemistry, Allegheny College, Meadville, Pennsylvania 16335, United States
