# Supplementary material for: Substituent Effects Govern the Efficiency of Isoxazole Photoisomerization to Carbonyl‑2H‑Azirines
Source: ACS Org Inorg Au. 2025 Dec 1;6(1):41–6. doi: 10.1021/acsorginorgau.5c00105 (PMC12879163; doi:10.1021/acsorginorgau.5c00105)
Supplement: Supplementary file 1 [file gg5c00105_si_001.pdf]

SUPPORTING INFORMATION FOR

Substituent Effects Govern the Efficiency of Isoxazole Photoisomerization to Carbonyl-2*H*-Azirines

Kyra E. Jackson[1,2], Isabelle Szeto[1], Leah M. Seebald[1]*, Samuel G. Shepard[1,3]*

[1] Department of Chemistry, Haverford College, Haverford, PA 19041, United States
[2] Current Affiliation: Department of Chemistry, University of Chicago, Chicago, IL 60637, United States
[3] Current Affiliation: Department of Chemistry, Allegheny College, Meadville, PA 16335, United States
*Co-corresponding authors: lseebald@haverford.edu, sshepard@allegheny.edu

---

TABLE OF CONTENTS

EXPERIMENTAL METHODS 3

Figure S1 3

COMPUTATIONAL METHODS 4

Figure S2 4

Figure S3 5

Figure S4 5

REACTION MONITORING 6

Figure S5 6

Figure S6 7

Figure S7 7

Figure S8 8

Figure S9 9

Figure S10 10

SYNTHESIS OF ISOXAZOLES 11

General procedure for the synthesis of isoxazoles (Is-1 and Is-2) 11

3-phenyl-5-isoxazolamine (Is-1) 11

3-tert-butyl-5-isoxazolamine (Is-2) 11

PHOTOISOMERIZATION OF ISOXAZOLES 12

General procedure for the small-scale photoisomerization of isoxazoles 12

3-phenyl-2H-azirine-2-carboxamide (Az-1) 12

3-(tert-butyl)-2H-azirine-2-carboxamide (Az-2) 12

3-(trifluoromethyl)-2H-azirine-2-carboxamide (Az-3) 13

General procedure for the scaled-up photoisomerization of isoxazoles *13*

Scaled-up synthesis of Az-2 .... *13*

Scaled-up synthesis of Az-3 .... *13*

^{1}H NMR SPECTRA 15

^{19}F NMR SPECTRA 20

ADDITIONAL COMPUTATIONAL DATA 21

Cartesian coordinates of optimized geometries *21*

Excitation energies, oscillator strengths, and orbital character for transitions *21*

Table S1 25

Table S2 26

Table S3 27

Comparison of extra diffuse basis set *28*

Figure S17 28

Figure S18 28

Figure S19 29

Relative energies of simulated species *29*

Table S4 29

Population analysis *29*

Table S5 30

REFERENCES 31

EXPERIMENTAL METHODS

All chemicals were purchased from commercial vendors and used without further purification unless indicated otherwise. Reaction progress was monitored by TLC on pre-coated silica plates (Miles Scientific F254 nm, 200 μm) and spots were visualized by UV. Flash column chromatography was carried out using silica gel (SilicaFlash P60, 40–63 μm). All UV–vis samples were prepared using HPLC-grade solvents and spectra were obtained using an Agilent Cary 3500 spectrophotometer with 1 cm path length quartz cuvettes (3-Q-10-GL14-S, Starna Cells). Irradiation was carried out using a medium power (51 mW) 255 (±5) nm UVC LED chip-on-board package with a 13 nm full width at half maximum (FWHM) from Boston Electronics, which was mounted to a small heat sink.

Chemical shifts (δ) are reported as measured by a Bruker 400 MHz NMR spectrometer. Chemical shifts (δ) are reported in parts per million (ppm) and referenced with respect to residual deuterated solvent (CDCl3, δ = 7.26 ppm or CD3CN, δ = 1.94 ppm). Data are reported as follows: chemical shift (ppm), multiplicity (s = singlet, d = doublet, t = triplet, q = quartet, quin = quintet, sx = sextet, m = multiplet, dd = doublet of doublets, dt = doublet of triplets, dm = doublet of multiplets, brs = broad singlet), coupling constant (Hz), and integrations (#H). Spectral analysis was conducted using MestReNova.

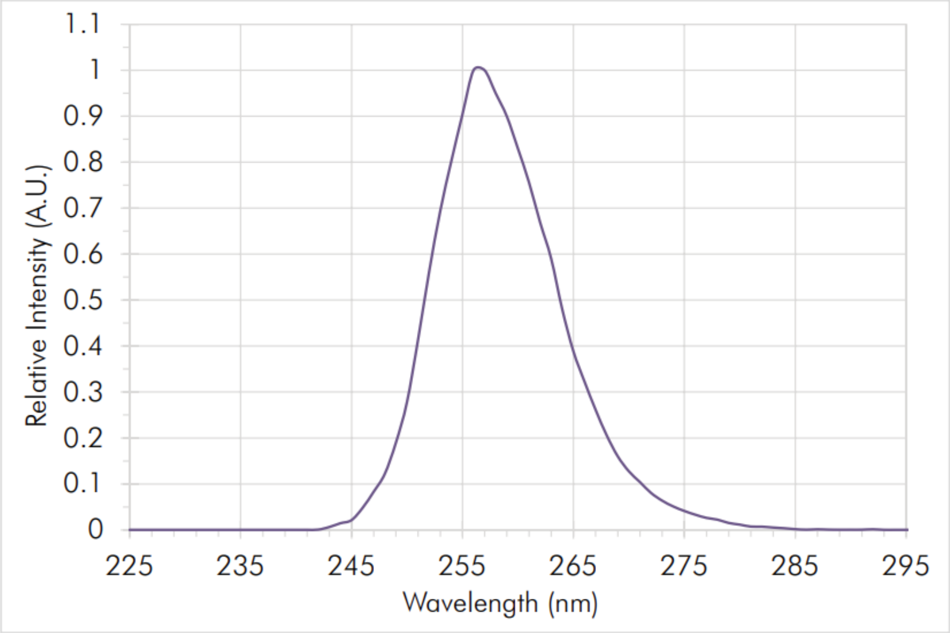

Figure S1. Emission spectrum of LED used for photoisomerization experiments. Reprinted from source.[1]

COMPUTATIONAL METHODS

Electronic structure calculations were performed in Gaussian 03[2] using WebMO. Structures were optimized at the B3LYP[3–6] /6-31G(d)[7,8] level of theory. Prior to TD-DFT, a vibrational analysis was run to confirm the absence of any imaginary vibrational frequencies. TD-DFT was performed at the TDB3LYP/6-311+G(2d,p)[9] with solvent (acetonitrile) simulated using a polarizable continuum model.[10] For all TD-DFT calculations, the lowest 10 roots were calculated. Unless otherwise noted, simulated spectra 4 were generated assuming a Gaussian line shape with a 0.4 eV FWHM. Orbitals were visualized in Avogadro.[11,12]

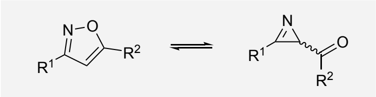

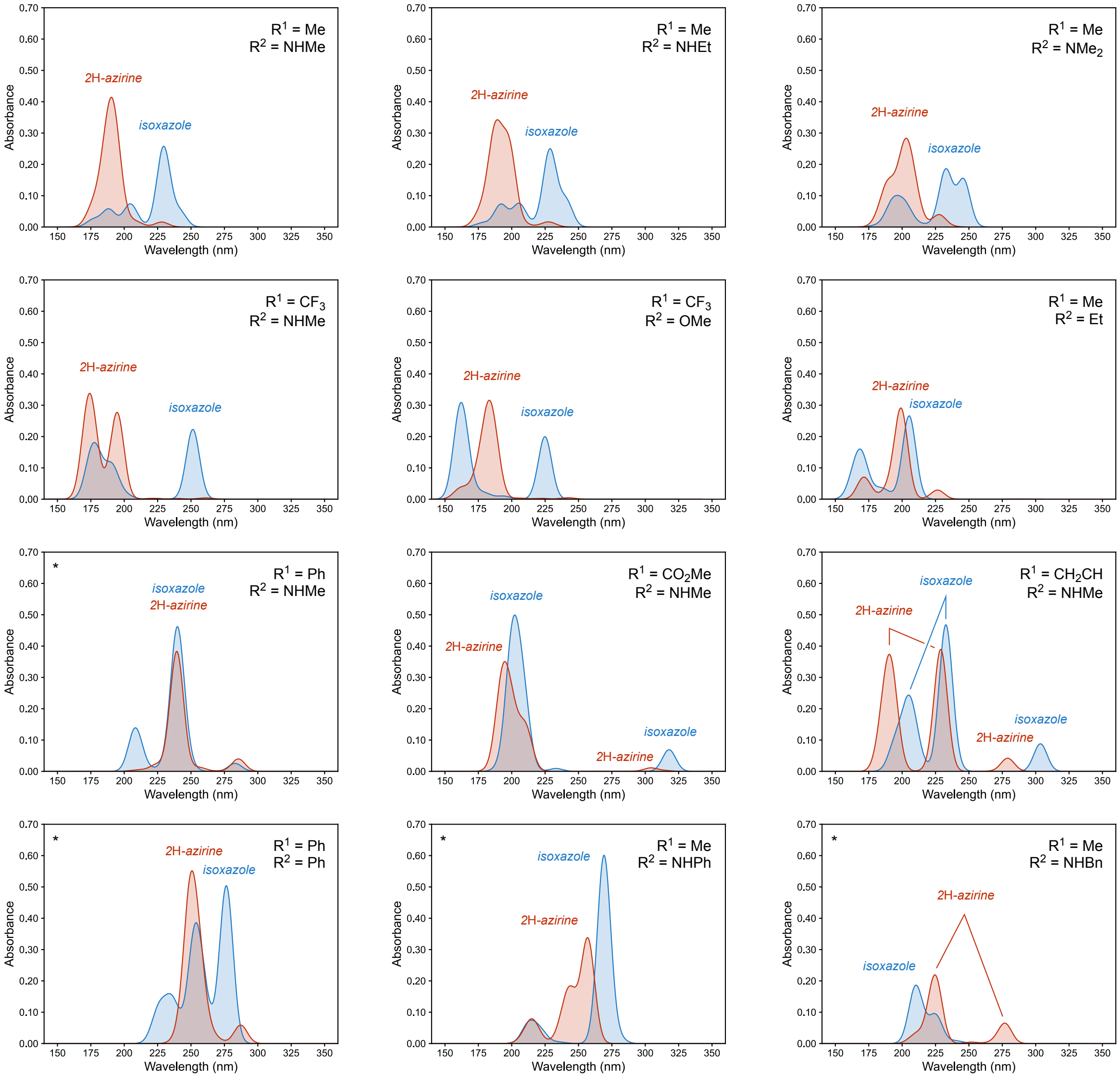

Figure S2. Simulated theoretical spectra for isoxazole–carbonyl-2H-azirine isomer sets show significant substituent dependence. All calculations were done at the TD-B3LYP/6-311+G(2d,p)+PCM(MeCN)//B3LYP/6-31G(d) level of theory. Plots marked with * were calculated without a solvation model. Unlike the simulated spectra in S3 and S4, these spectra were generated assuming a Gaussian lineshape and a 10 nm FWHM, so they underestimate any broadening on the red edge of the spectrum.

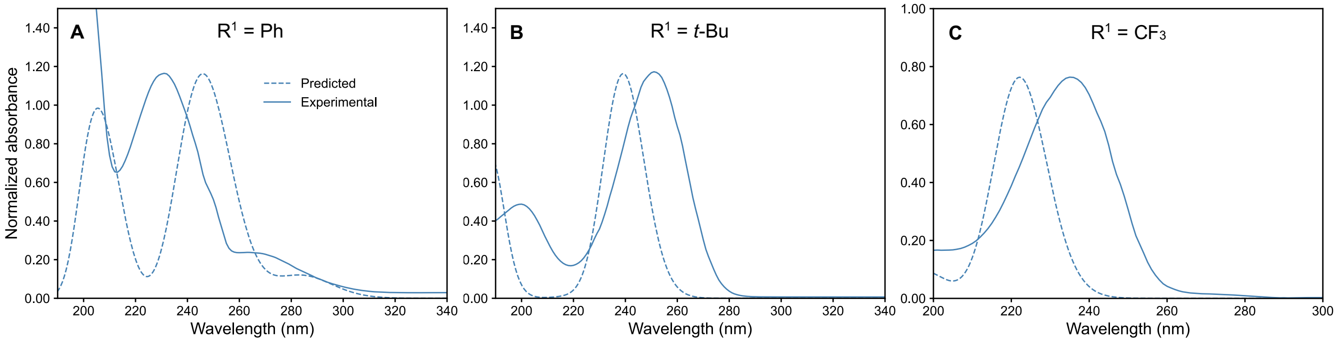

Figure S3. Comparison of calculated TD-DFT absorption spectra with experimental measurements for isoxazoles **Is-1** (**A**), **Is-2** (**B**), **Is-3** (**C**).

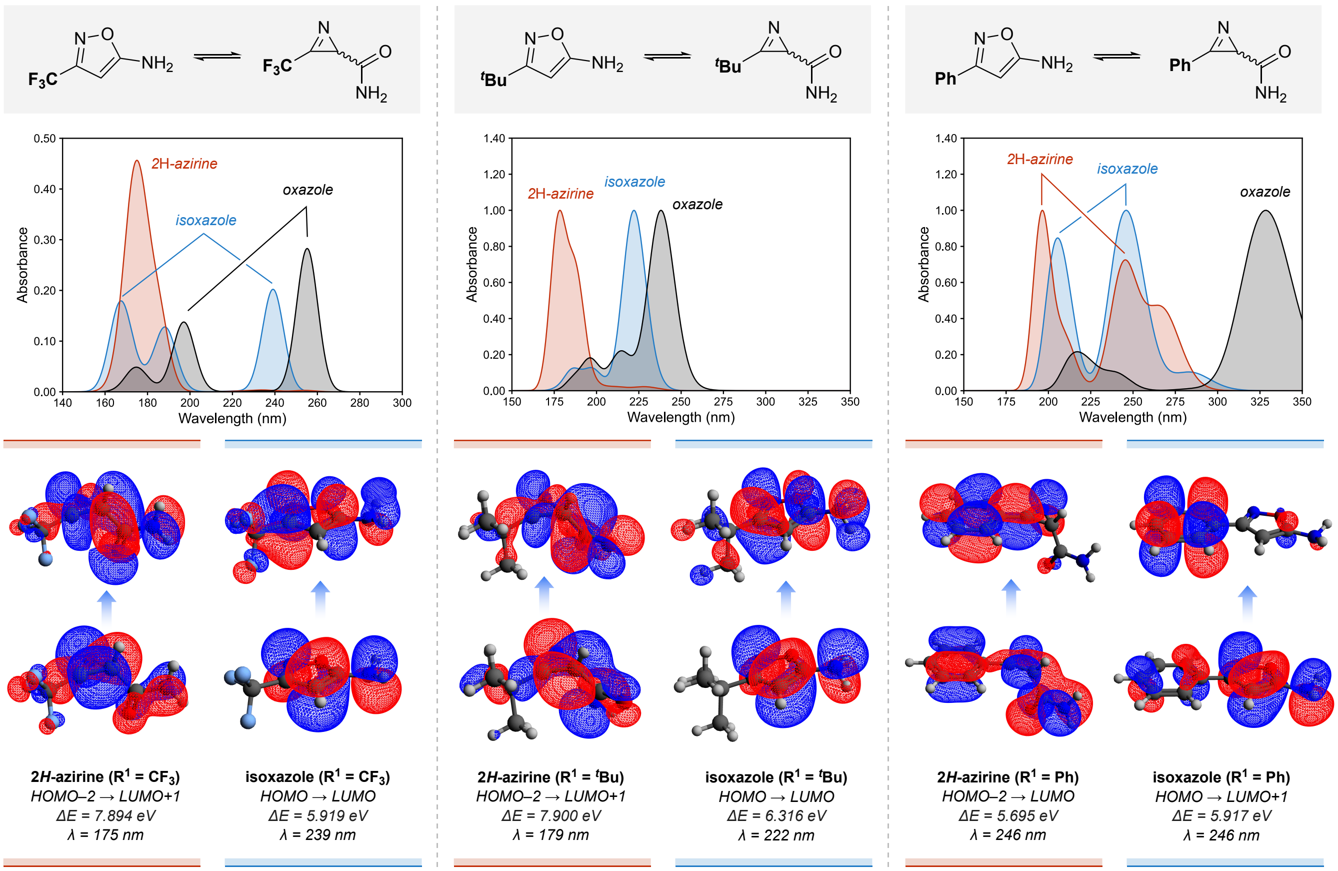

Figure S4. Isoxazole, 2*H*-azirine, and oxazole simulated absorption spectra calculated at the TD-B3LYP/6-311+G(2d,p)+PCM(MeCN)//B3LYP/6-31G(d) level of theory and relevant orbital transitions for trifluoromethyl-, tertbutyl-, and phenyl-substituted isoxazole–azirine isomer pairs. The orbitals shown are the primary electronic transitions occurring with irradiation at wavelengths relevant to photoisomerization studies.

REACTION MONITORING

UV–vis monitoring: Stock solutions of isoxazoles were prepared at 1 mg/mL concentrations in MeCN. From this stock solution, 0.5 µL was added into a cuvette with approximately 3 mL of HPLC-grade MeCN. The light source was positioned about 3 cm from the cuvette and the sample was irradiated, collecting UV–vis scans at regular intervals.

NMR monitoring: For NMR-scale photolysis, approximately 10 mg of isoxazole was dissolved in approximately 1 mL of CD_3CN in a cuvette. At this higher concentration, the light source is substantially attenuated by the highly concentrated sample, so the LED was positioned about 1 cm from the cuvette. The solution was irradiated and periodically transferred to an NMR tube to collect a spectrum at that time point. After NMR analysis, the sample was transferred back to the cuvette and irradiated further, repeating NMR experiments at desired time intervals. Again, due to the higher concentration of the sample for NMR-scale experiments, these reactions tended to take 10–30 times longer. These reactions were carried out at both rt and 4 °C (cold room) with no difference in conversion. Simultaneous monitoring of the NMR-scale reaction by UV–vis could be performed by taking small aliquots (0.5 µL) of the reaction mixture and diluting in 3 mL of HPLC-grade MeCN.

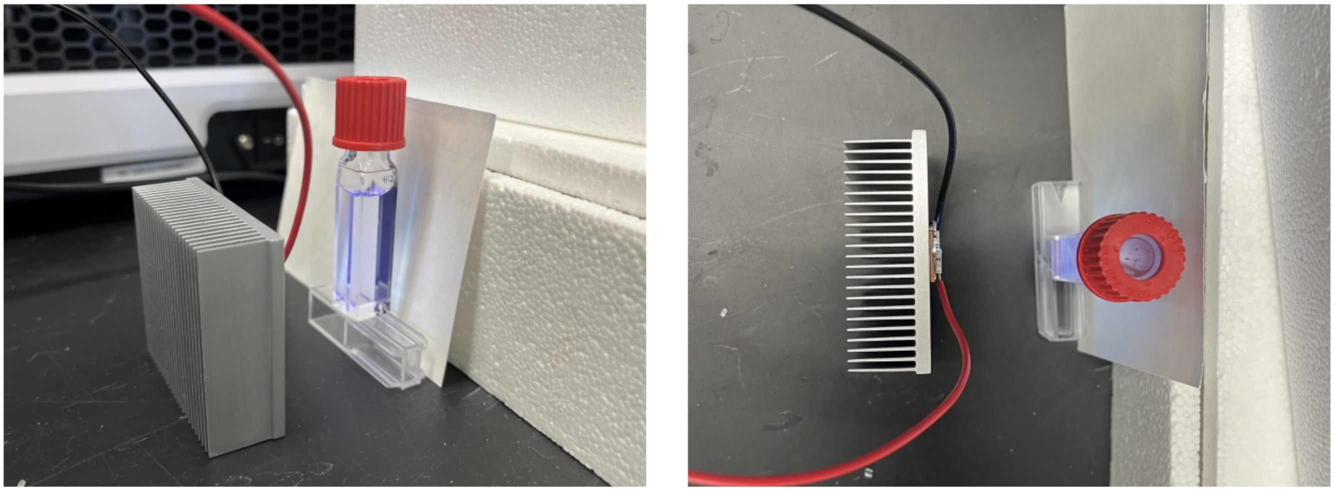

Figure S5. Photoisomerization setup: Pictured is a quartz cuvette (3-Q-10-GL14-S, Starna Cells) illuminated by 51 mW 255 nm LED (Boston Electronics). The cuvette was elevated to ensure illumination in the center of the solution.

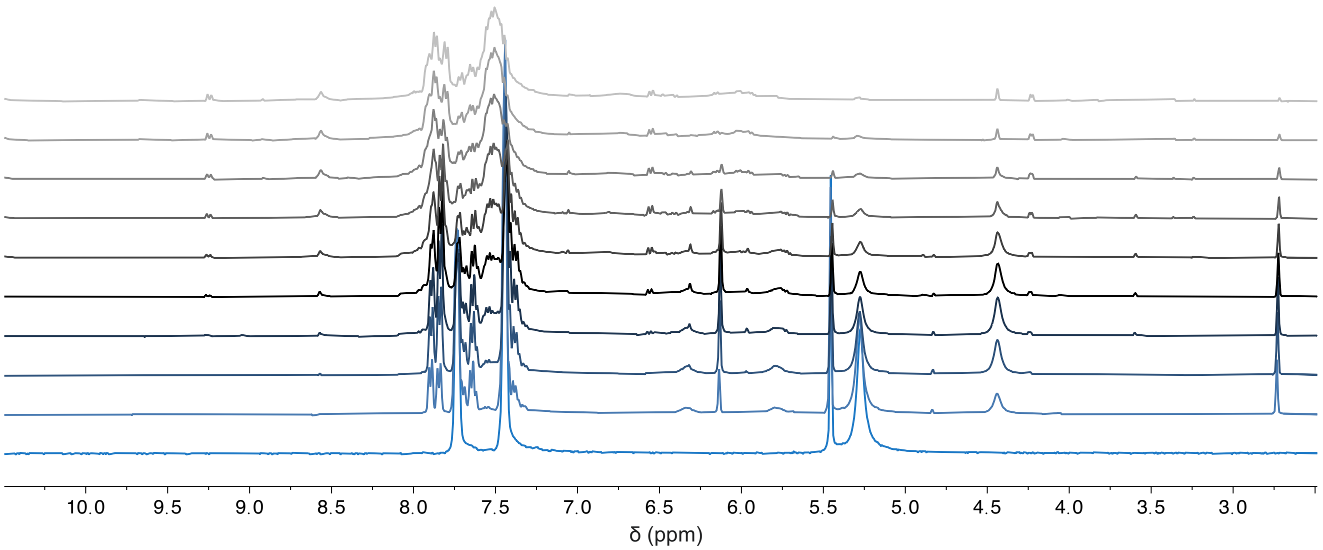

Figure S6. Full stacked ^{1}H NMR spectra (CD_3CN) of the photolysis of **Is-1**, yielding **Az-1** and **Ox-1**. Each spectrum is taken after an additional twenty minutes of irradiation; the data show changes over the course of three hours. The bottommost spectrum is **Is-1** at time $t = 0$ (CD_3CN, 400 MHz): δ 7.73 (d, $J = 3.19$ Hz, 2H), 7.44 (s, 3H), 5.45 (s, 1H), 5.27 (brs, 2H).

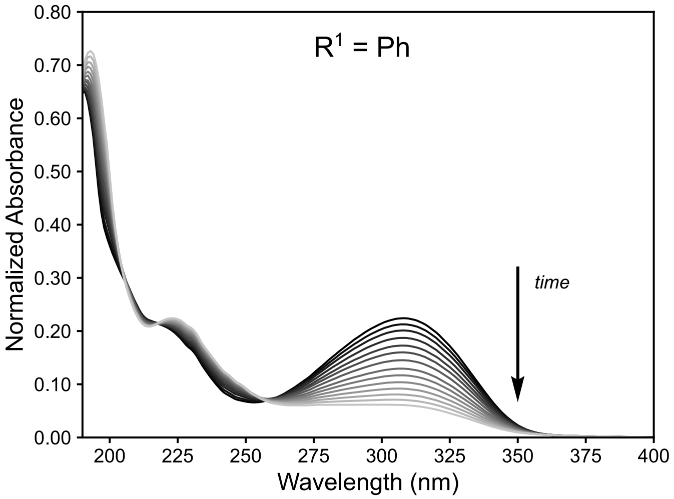

Figure S7. UV–vis data for photolysis of **Is-1** beyond the initial minute shown in **Figure 2B**. Spectra collected between 1–8 minutes of illumination show the characteristic absorption band of the oxazole centered at 310 nm decaying away.

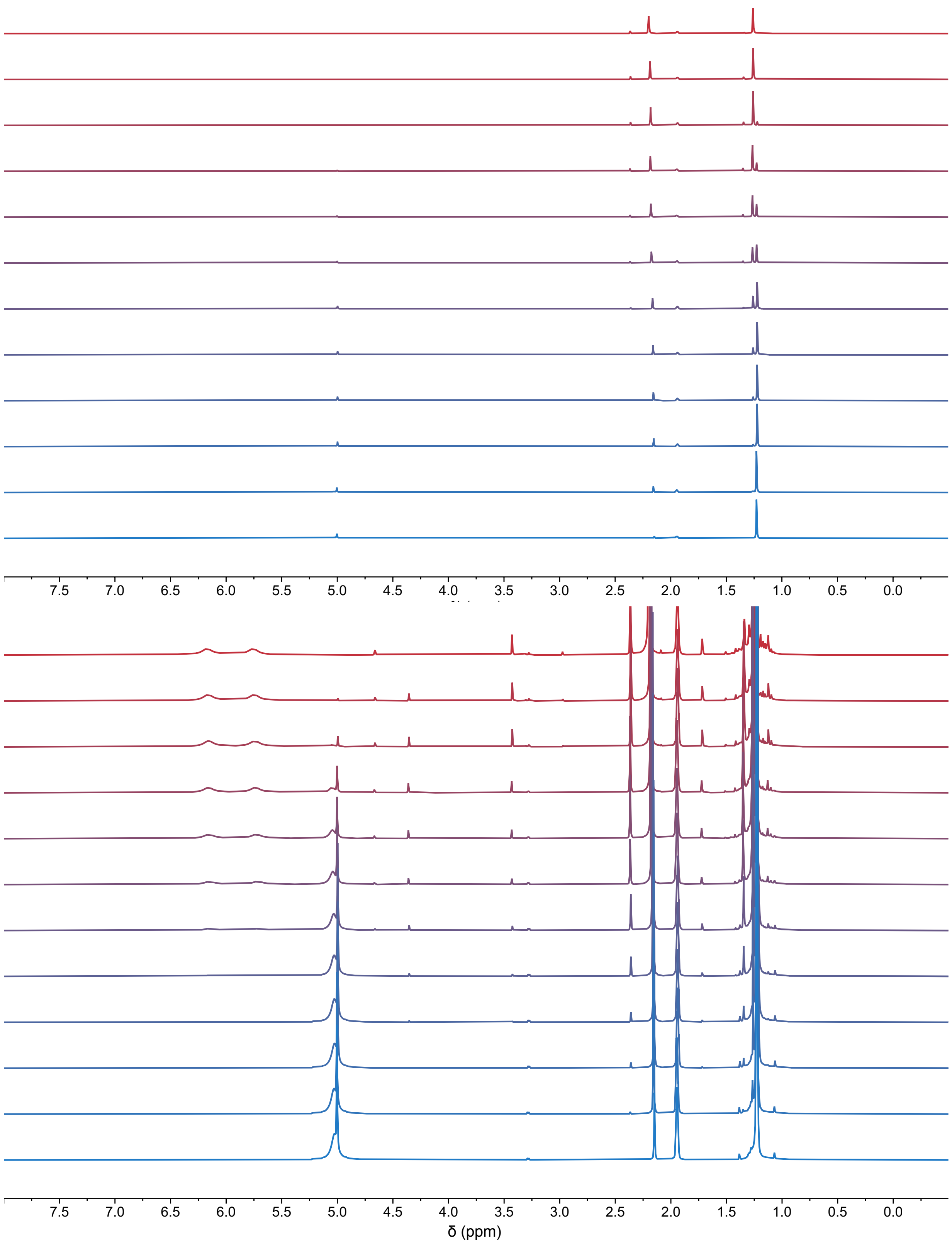

Figure S8. Full stacked ^{1}H NMR spectra. Top: vertically zoomed in for identification of small intensity peaks. Bottom: full spectrum (vertically zoomed out relative to above) of the photolysis of **Is-2** in CD_3CN, yielding **Az-2.** Spectra were collected at the following time points: 0, 2, 4, 10, 20, and 40 minutes, 1, 1.5, 2, 3, 4, and 8 hours. For both stacks, the bottommost spectrum is **Is-2** at time t = 0 (CD_3CN, 400 MHz): δ 5.02 (brs, 2H), 5.00 (s, 1H), 1.22 (s, 9H).

The following unassigned or notable peaks in **Figure S8** are included here for reference:

δ 5.02 ppm: The amine peak of the isoxazole is downshifted in CD_3CN (5.02 ppm) relative to in $CDCl_3$ (4.34 ppm).

δ 3.42/1.72 ppm: A pair of sharp singlets that emerge upon carbonyl-2*H*-azirine formation. These peaks show up in a 1:1 ratio relative to each other, and a 0.25:1 ratio relative to the 2*H*-azirine proton. These peaks emerge across multiple replicates and scales. They have not been definitively assigned.

δ 2.15 ppm: H_2O.

δ 1.95 ppm: Residual signal from CD_3CN (solvent).

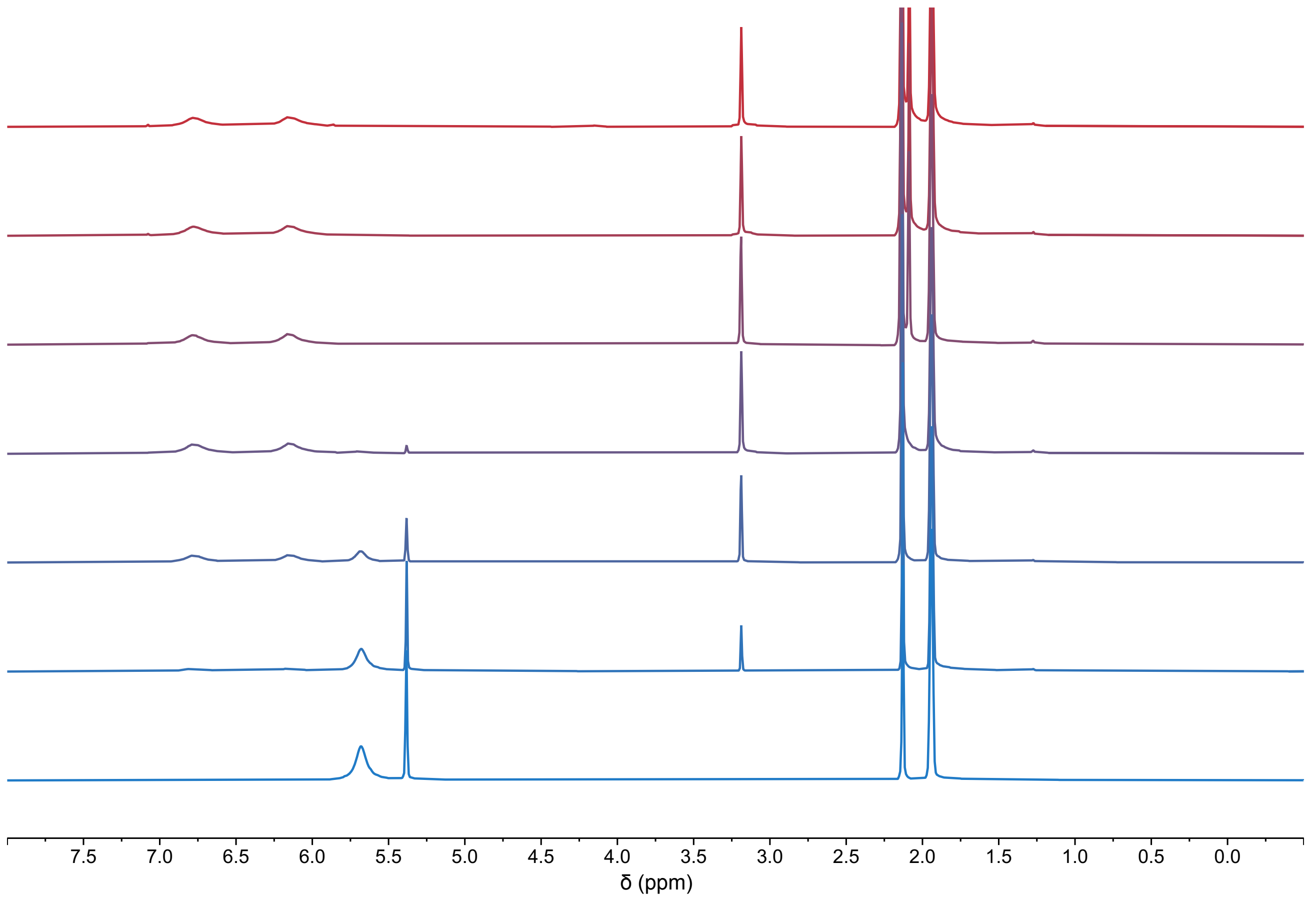

Figure S9. Full stacked ^{1}H NMR of photoisomerization of **Is-3** in CD_3CN, yielding **Az-3**. Also present are impurities of water, acetone, and the residual solvent. Each spectrum is taken after an additional 5 minutes of irradiation; the data show changes over the course of 30 minutes. The bottommost spectrum is **Is-3** at time t = 0 (CD_3CN, 400 MHz): δ 5.68 (brs, 2H), 5.38 (s, 1H).

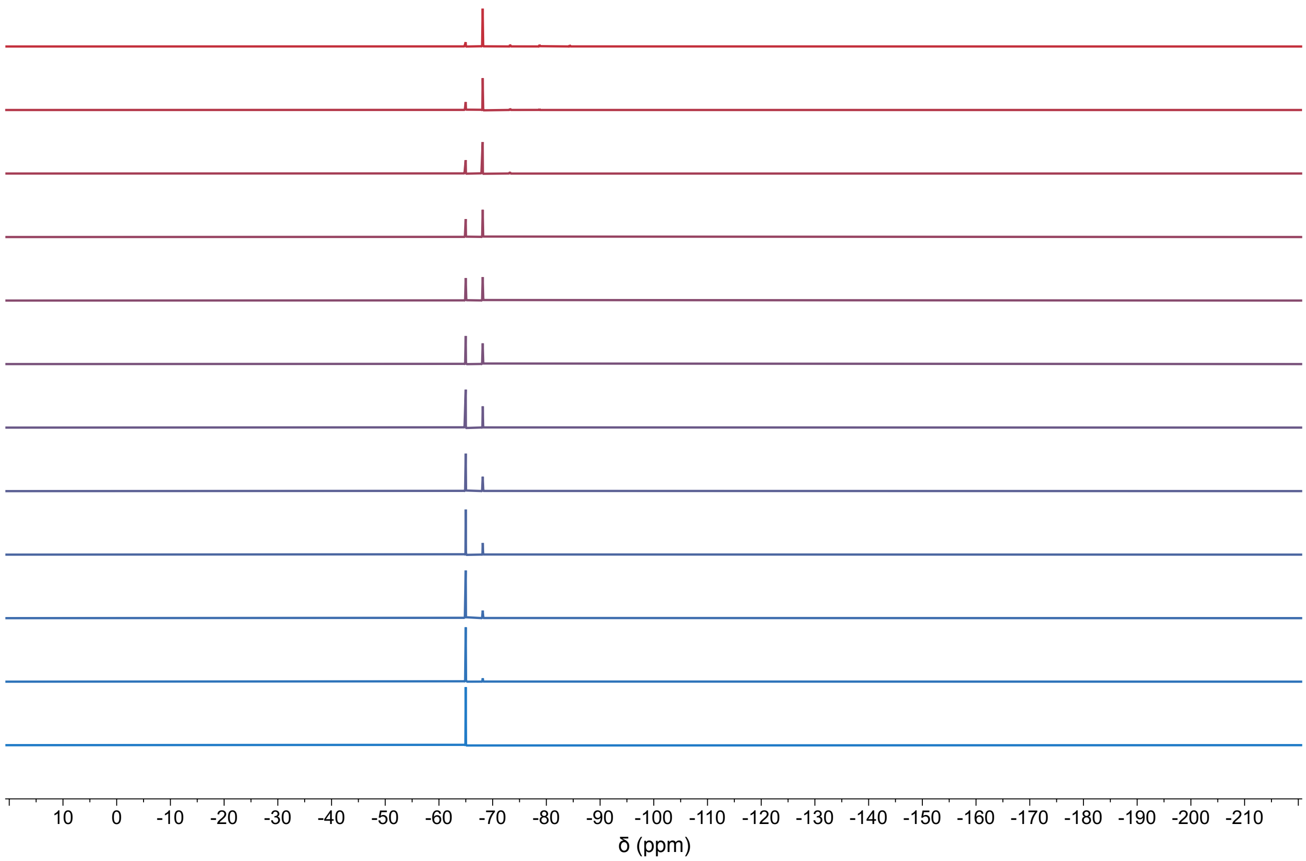

Figure S10. Full stacked ^{19}F NMR of photoisomerization of **Is-3** in CD_3CN, yielding **Az-3**. Each spectrum is taken after an additional 2 minutes of irradiation; the data show changes over the course of 24 minutes. The bottommost spectrum is **Is-3** at time *t* = 0 (CD_3CN, 400 MHz): δ –65.0.

SYNTHESIS OF ISOXAZOLES

General procedure for the synthesis of isoxazoles (Is-1 and Is-2)

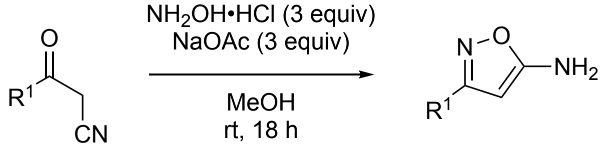

Is-1, R^1 = Ph
Is-2, R^1 = *t*-Bu

The following protocol was adapted from a previously reported method.[13] In a small round bottom flask, $NH_2OH{\cdot}HCl$ (3 equiv) and NaOAc (3 equiv) were stirred in anhydrous MeOH (15 mL) at rt for 1 h before the nitrile (1 equiv) was added to the mixture. The reaction stirred at rt for 18 h. Most of the MeOH solvent was removed *in vacuo*, and the residue was resuspended in water, extracted with EtOAc, and washed with brine. The organic phase was dried over $MgSO_4$ and concentrated *in vacuo*. No further purification was performed. Note: If trace amounts of acetic acid are observed by NMR, additional washes with saturated Na_2CO_3 can be used to remove these traces.

3-phenyl-5-isoxazolamine (Is-1)

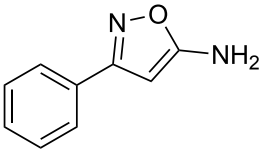

Following the general procedure for isoxazole synthesis with NH2OH·HCl (1.04 g, 15.0 mmol, 3 equiv), NaOAc (1.23 g, 15.0 mmol, 3 equiv) and benzoyl-acetonitrile (0.72 g, 5.0 mmol, 1 equiv), **Is-1** was obtained as a crystalline, pale orange (peach-colored) solid (0.64 g, 80% yield), consistent with previously reported data[14] (**6k** in referenced paper). ^{1}H NMR (CDCl3, 400 MHz) δ 7.73 (s, 2H), 7.42 (s, 3H), 5.45 (s, 1H), 4.48 (brs, 2H).

*3-*tert*-butyl-5-isoxazolamine (Is-2)*

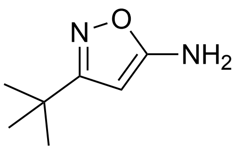

Following the general procedure for isoxazole synthesis with NH2OH·HCl (1.04 g, 15.0 mmol, 3 equiv), NaOAc (1.23 g, 15.0 mmol, 3 equiv) and trimethyl-acetylacetonitrile (0.66 g, 5 mmol, 1 equiv), **Is-2** was obtained as a crystalline, yellow solid (0.57 g, 81% yield), consistent with previously reported data[14] (**6j** in referenced paper). ^{1}H NMR (CDCl3, 400 MHz) δ 5.03 (d, J = 2.8 Hz, 1H), 4.32 (brs, 2H), 1.28 (s, 9H).

PHOTOISOMERIZATION OF ISOXAZOLES

General procedure for the small-scale (~10 mg/mL) photoisomerization of isoxazoles to carbonyl-2*H*-azirines

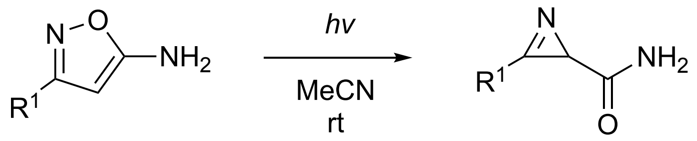

Approximately 30 mg of isoxazole was dissolved in about 3 mL of MeCN in a quartz cuvette (10 mg/mL, 0.06–0.07 mM). The light source was positioned about 1 cm from the cuvette and the sample was irradiated with a 255 nm LED. When samples were monitored by NMR, isoxazole samples were dissolved and irradiated directly in MeCN-d_3. Approximate scale and time to reach completion are reported for each carbonyl-2*H*-azirine below.

*3-phenyl-2*H*-azirine-2-carboxamide (Az-1)*

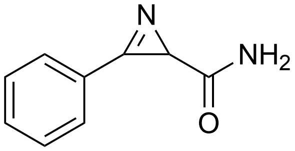

Following the "general procedure for the small-scale (~10 mg/mL) photoisomerization of isoxazoles to carbonyl-2*H*-azirines," **Az-1** did not cleanly photoisomerize from **Is-1** (31 mg, 0.19 mmol). The NMR presents a mixture of **Is-1**, **Az-1**, and **Ox-1**. Attempts to photoisomerize **Is-1** completely resulted in driving **Az-1** to **Ox-1** and further degradation products. Irradiation was stopped upon full consumption of **Is-1** as monitored by UV-vis. **Az-1** chemical shifts matched with current literature[15] (**2f** from referenced paper). The resultant crude material was obtained as a hygroscopic off-white solid. Yield is reported after full conversion of **Is-1** was observed (23 mg, 74%).

Ox-1: Phenyl region unassigned; overlaps with **Is-1** and **Az-1**. ^{1}H NMR (400 MHz, CD_3CN) δ 6.14 (s, 1H, ring), 4.44 (s, 2H, amine).

Az-1: Phenyl region unassigned; overlaps with **Is-1** and **Ox-1**. ^{1}H NMR (400 MHz, CD_3CN) δ 6.32 (s, 1H, amide), 5.80 (s, 1H, amide), 2.74 (s, 1H, 2*H*-azirine).

*3-(*tert*-butyl)-2*H*-azirine-2-carboxamide (Az-2)*

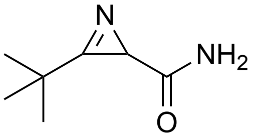

Following the "general procedure for the small-scale (~10 mg/mL) photoisomerization of isoxazoles to carbonyl-2*H*-azirines," **Is-2** (27 mg, 0.19 mmol) underwent photoisomerization to give **Az-2**. The reaction was stopped when quantitative conversion of **Is-2** to **Az-2** was observed (**2d** from referenced paper).[15] **Az-2** was obtained as a powdery orange solid, and the calculated percent yield from this small-scale reaction was comparable to that of the scaled-up reaction described in the following section (17 mg, 87%). ^{1}H NMR (400 MHz, CD_3CN) δ 5.95 (d, J = 166.1 Hz, 2H), 2.36 (s, 1H), 1.26 (s, 9H). When carried out at approximately 4 µg/mL, the reaction reaches completion at 20 min. When carried out at 10 mg/mL, the reaction reaches completion at 4 h. When scaled up to 25 mg/mL, the reaction reaches completion in 10 h.

*3-(trifluoromethyl)-2*H*-azirine-2-carboxamide (Az-3)*

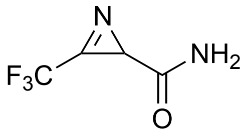

Following the "general procedure for the small-scale (~10 mg/mL) photoisomerization of isoxazoles to carbonyl-2*H*-azirines," Is-3 (35 mg, 0.23 mmol) underwent photoisomerization to give **Az-3**. The reaction was stopped when quantitative conversion **Is-3** to **Az-3** was observed. The calculated percent yield from this small-scale reaction was comparable to that of the scaled-up reaction described in the following section; little material is left after solvent removal (0.9 mg, 3%). This amount was too small to permit adequate visual characterization and is therefore better described in the scaled-up reaction in the following section. 1H NMR (400 MHz, CD_3CN) δ 6.78 (s, 1H), 6.17 (s, 1H), 3.21 (s, 1H). 19F NMR (400 MHz, CD_3CN) –68.14 ppm. When carried out at approximately 4 µg/mL, the reaction reaches completion at 1 min. When carried out at 10 mg/mL, the reaction reaches completion at 30 min.

*General procedure for the scaled-up photoisomerization (~25 mg/mL) of isoxazoles to carbonyl-2*H*-azirines*

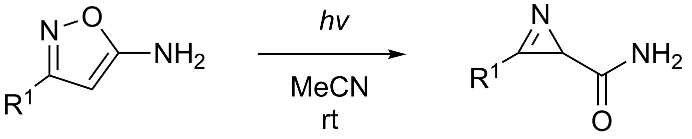

Az-1, R^1 = Ph
Az-2, R^1 = *t*-Bu
Az-3, R^1 = CF_3

Approximately 100 mg of isoxazole was dissolved in about 4 mL of MeCN in a quartz cuvette (~25 mg/mL, 0.16–0.18 mM). The light source was positioned about 1 cm from the cuvette and the sample was irradiated with a 255 nm LED. The reaction was monitored by TLC coupled with either UV–vis and/or NMR for completion. When samples were monitored by NMR, isoxazole samples were dissolved and irradiated directly in MeCN-d_3. Approximate scale and time to reach completion are reported for each carbonyl-2*H*-azirine below.

Scaled-up synthesis of Az-2

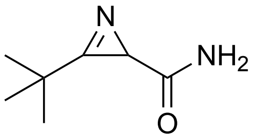

Following the "general procedure for the scaled-up photoisomerization (~25 mg/mL) of isoxazoles to carbonyl-2*H*-azirines," **Is-2** (100 mg, 0.71 mmol) underwent photoisomerization to give **Az-2**. The reaction was stopped when quantitative conversion of **Is-2** to **Az-2** was observed. Reaction reached completion after 10 h. After solvent was removed *in vacuo*, **Az-2** was isolated as a crystalline orange solid with no further purification (88 mg, 87% yield). ^{1}H NMR (400 MHz, $CDCl_3$) δ 5.34 (s, 2H), 2.43 (s, 1H), 1.32 (s, 9H).

Scaled-up synthesis of Az-3

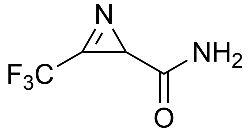

Following the "general procedure for the scaled-up photoisomerization (~25 mg/mL) of isoxazoles to carbonyl-2*H*-azirines," **Is-3** (97 mg, 0.64 mmol) underwent photoisomerization to give **Az-3**. The reaction was stopped when quantitative conversion of **Is-3** to **Az-3** was observed. Reaction reached completion

after 10 h, and ^{1}H NMR confirmed the **Az-3** structure. After removal of solvent *in vacuo*, the mass was significantly reduced (9.1 mg, 9% yield) and the sticky, white, semi-solid residue could not be solubilized or transferred easily by pipette. The subsequent ^{1}H and ^{13}C NMR spectra did not match **Az-3**, suggesting degradation and/or polymerization. The low isolated yield is likely due to the loss of lower-molecular-weight degradation products during evaporation. These results indicate that this particular trifluoromethyl 2*H*-azirine is too reactive to be isolated after solvent removal. Nonetheless, this method enables clean *in situ* generation of **Az-3** for immediate use, without the need for purification or isolation.

^{1}H NMR SPECTRA

Is-1 ^{1}H NMR (400 MHz, $CDCl_3$)

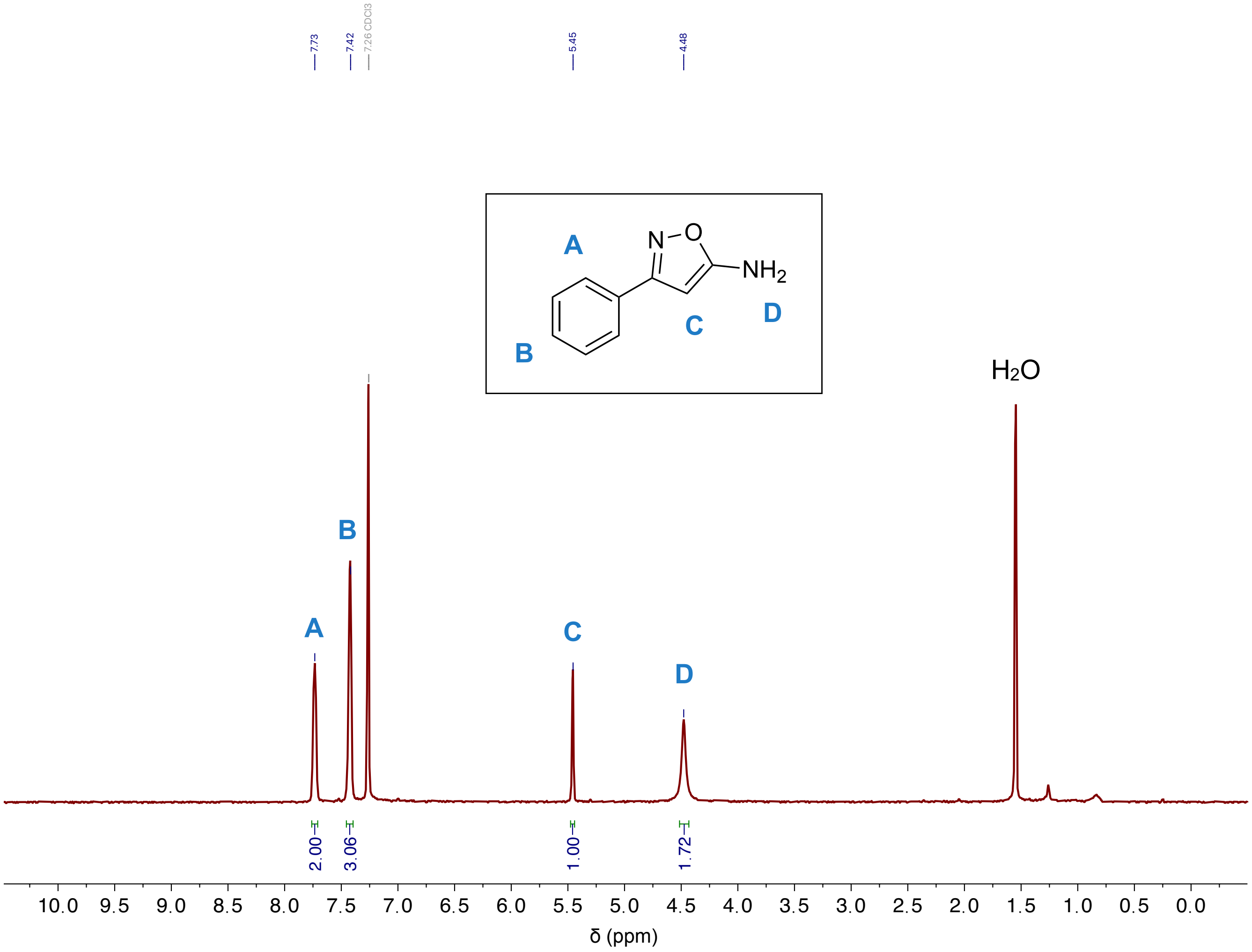

Is-2 ^{1}H NMR (400 MHz, $CDCl_3$)

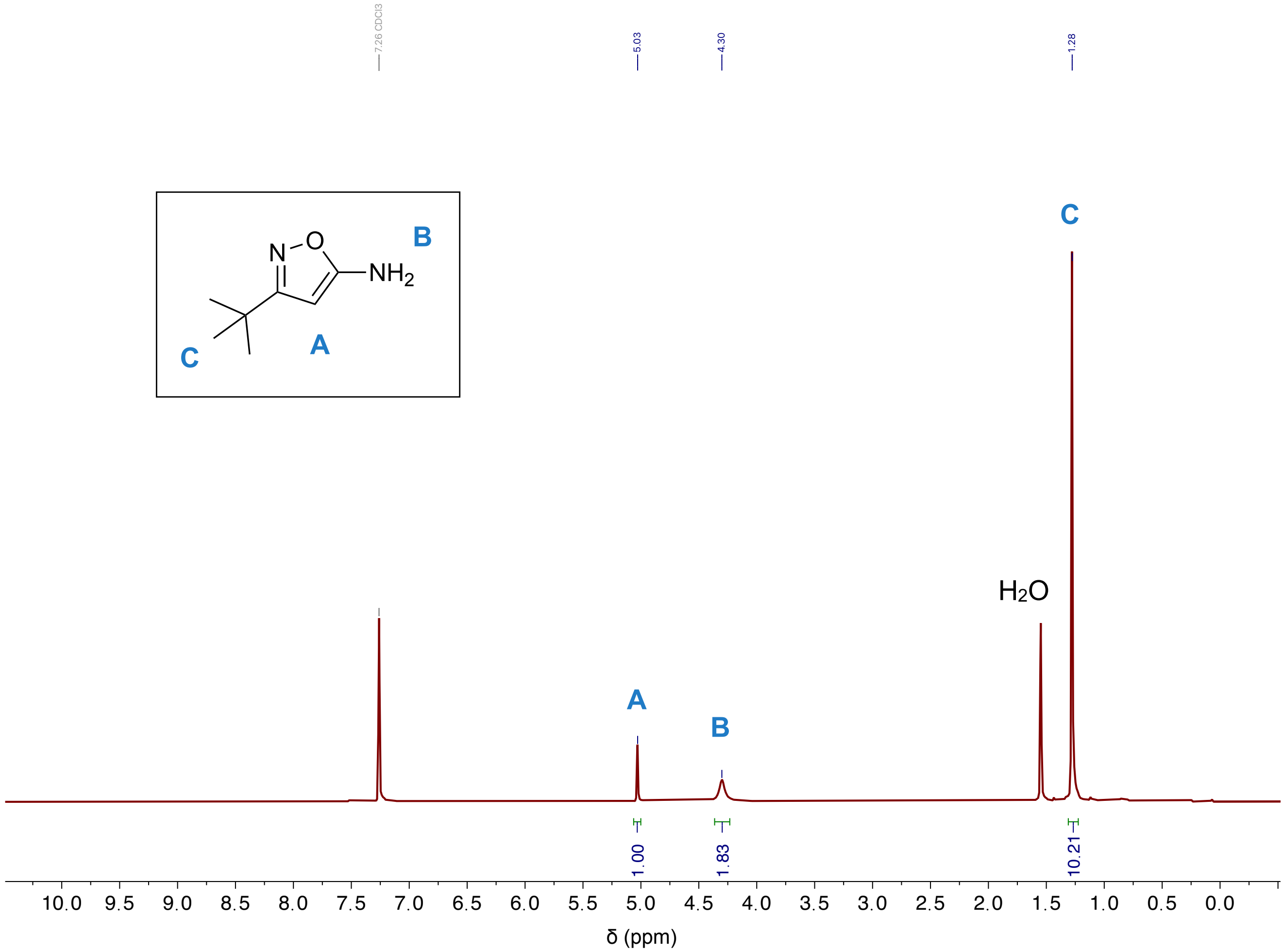

Az-1 ^{1}H NMR (400 MHz, CD_3CN) *

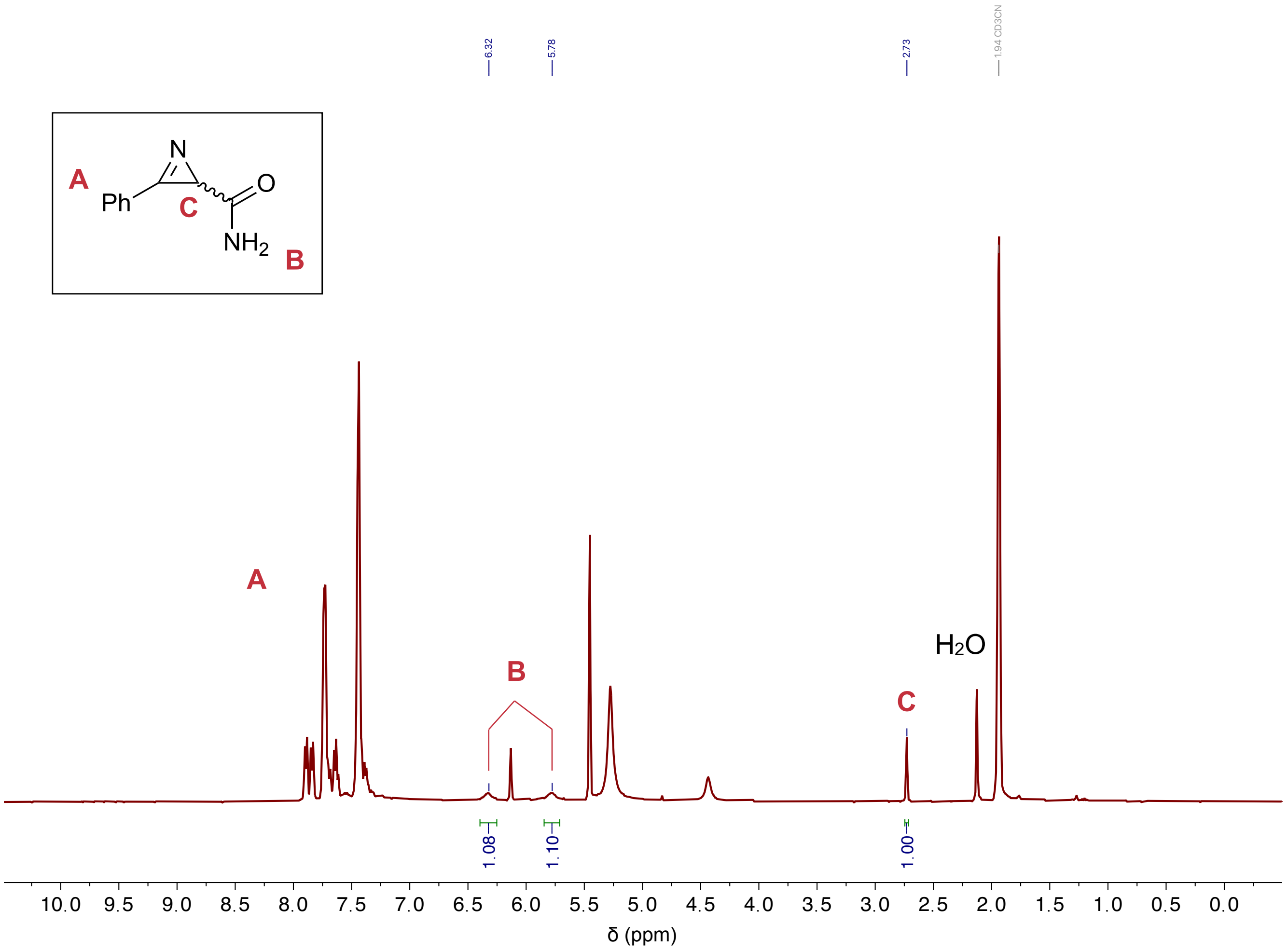

* The integration of A captures overlapping peaks of the isoxazole, oxazole, carbonyl-2*H*-azirine and any aromatic-containing byproducts and we cannot definitively integrate for just the 5H phenyl of the carbonyl-2*H*-azirine.

Az-2 ^{1}H NMR (400 MHz, CD_3CN)

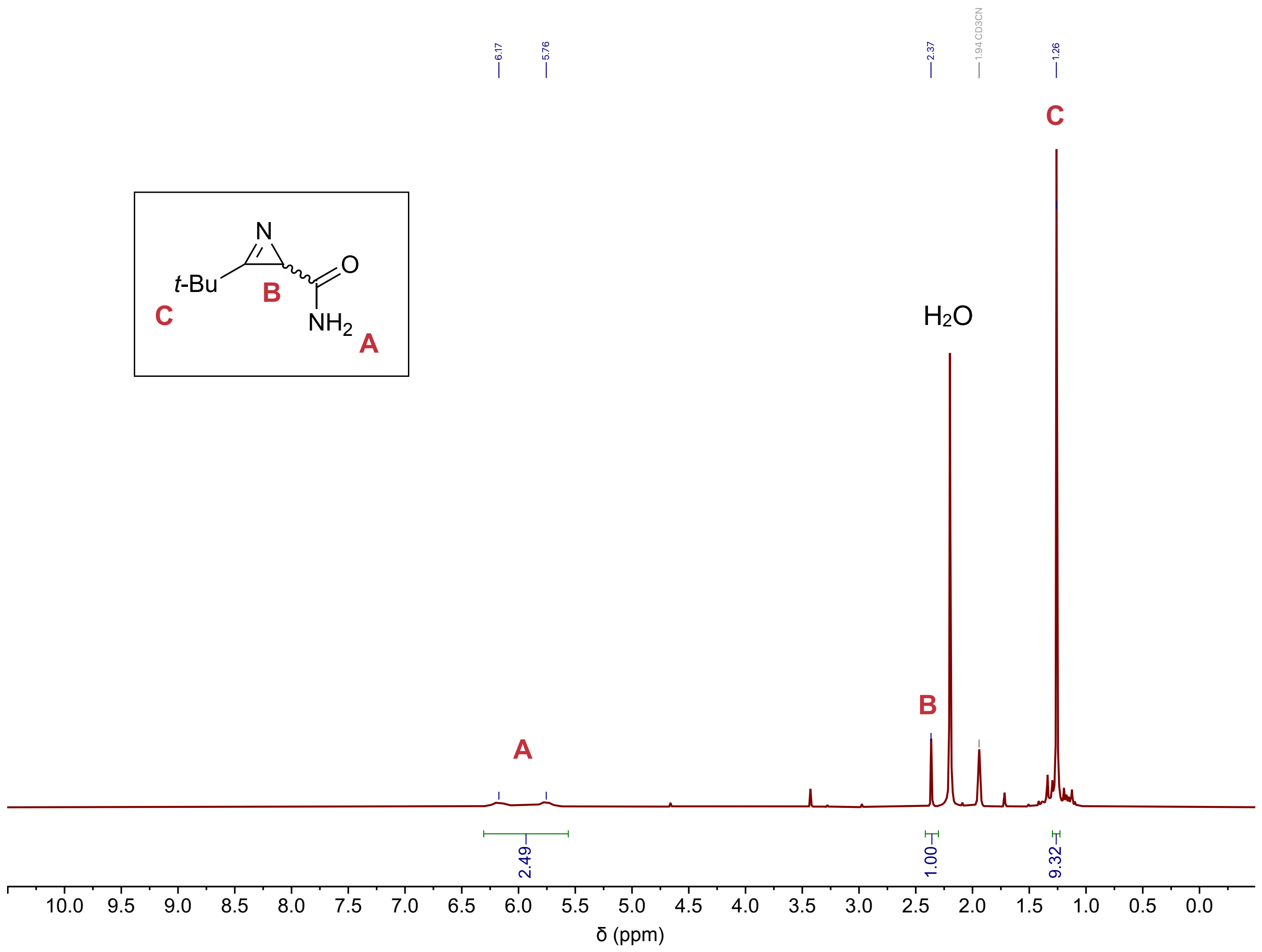

Az-3 ^{1}H NMR (400 MHz, CD_3CN)

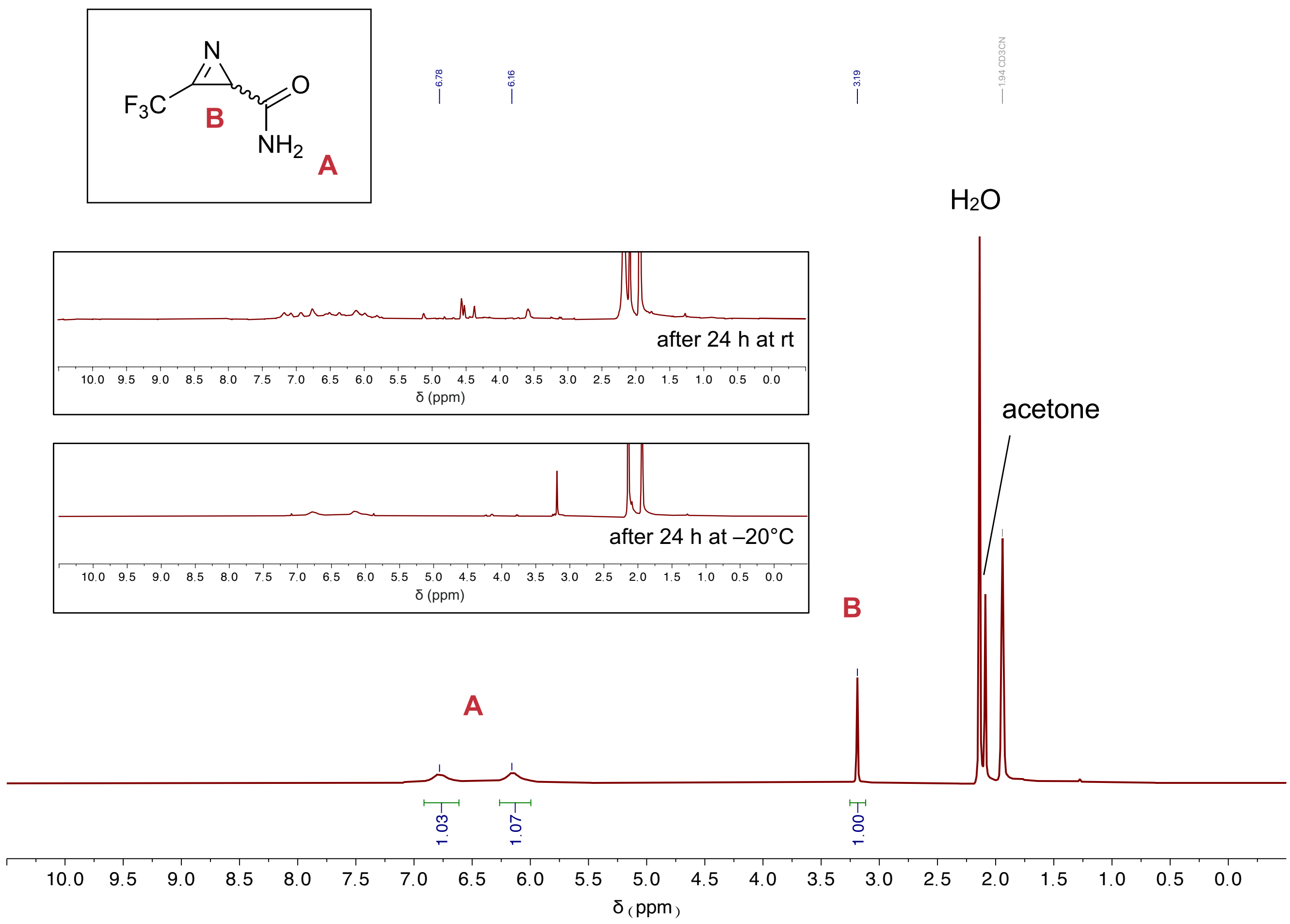

^{19}F NMR SPECTRA

Az-3 ^{19}F NMR (400 MHz, CD_3CN)*

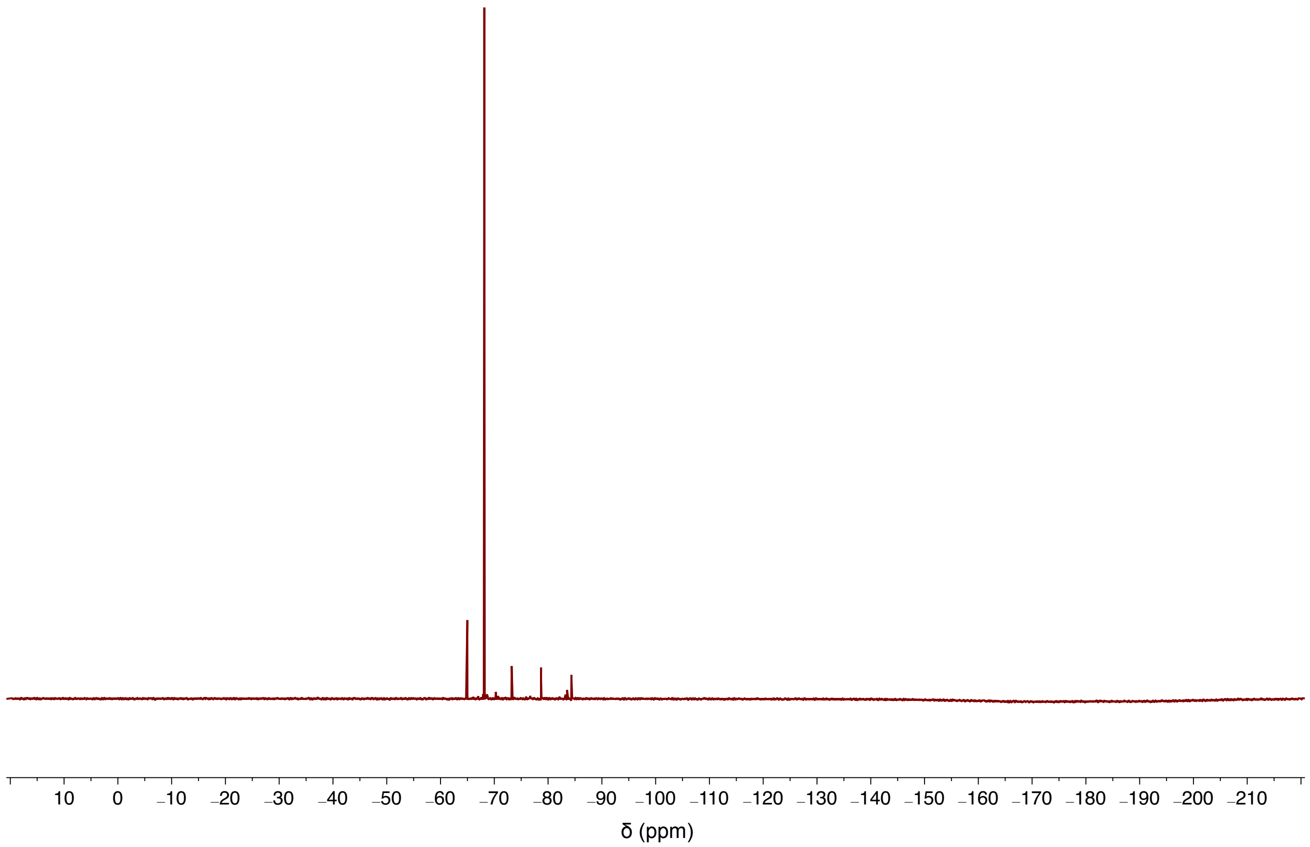

*Consistent with prior observations from scaled-up reactions, **Az-3** (–68.1 ppm) exhibits instability at room temperature, undergoing rapid decomposition over time. Decomposition can be slowed by generating the compound at lower temperature. In this ^{19}F NMR spectrum, degradation products started to emerge prior to reaction completion, so **Is-3** can still be seen (–65.0 ppm). These observations reflect the intrinsic instability of **Az-3** rather than limitations of the photoisomerization. For synthetic applications, **Az-3** should be generated immediately prior to use; prolonged storage, or isolation from solvent, is not recommended.

ADDITIONAL COMPUTATIONAL DATA

Cartesian coordinates of optimized geometries

3-phenyl-5-aminoisoxazole (Is-1)

N 0.0000000000 0.0000000000 0.0000000000
C 1.3121190000 -0.4094060000 0.0637550000
C 2.5089850000 0.2542190000 0.0660850000
C 3.4738630000 -0.7975610000 0.0681930000
N 2.9063880000 -1.9905880000 0.0693210000
O 1.5148720000 -1.7384340000 0.0679140000
C 4.9449920000 -0.6848380000 0.0702650000
C 5.7456510000 -1.8396030000 0.0743610000
C 7.1335040000 -1.7354570000 0.0763400000
C 7.7475230000 -0.4797710000 0.0744240000
C 6.9605220000 0.6721840000 0.0702620000
C 5.5696160000 0.5705660000 0.0681310000
H 4.9690240000 1.4754190000 0.0641570000
H 7.4279830000 1.6532660000 0.0682270000
H 8.8313720000 -0.4009560000 0.0758170000
H 7.7395410000 -2.6375620000 0.0792040000
H 5.2648520000 -2.8121720000 0.0753330000
H 2.6696140000 1.3206800000 0.0571900000
H -0.6659160000 -0.6907760000 0.3253900000
H -0.1768100000 0.9220430000 0.3765490000

3-*tert*-butyl-5-aminoisoxazole (Is-2)

C 0.0000000000 0.0000000000 0.0000000000
C 1.5062680000 0.1991390000 0.0015710000
C 2.5136580000 -0.8126990000 0.0082010000
C 3.6830870000 -0.1013090000 0.0107760000
O 3.4289830000 1.2175490000 0.0022610000
N 2.0185040000 1.4105950000 -0.0022020000
N 5.0113960000 -0.4595140000 0.0787090000
H 5.2233180000 -1.3736370000 -0.2993050000
H 5.6485060000 0.2561160000 -0.2508630000
H 2.3917210000 -1.8849780000 0.0194640000
C -0.3946770000 -0.8144230000 -1.2524940000
H -0.1156130000 -0.2845420000 -2.1699650000
H 0.0933670000 -1.7955260000 -1.2669460000
H -1.4788510000 -0.9779080000 -1.2698010000
C -0.3999340000 -0.7838580000 1.2703060000
H 0.0895010000 -1.7634470000 1.3113780000

H -0.1251480000 -0.2310550000 2.1752980000
H -1.4840580000 -0.9480700000 1.2866210000
C -0.7325670000 1.3544670000 -0.0179640000
H -0.4723750000 1.9360130000 -0.9075750000
H -1.8163120000 1.1882400000 -0.0171870000
H -0.4743130000 1.9579570000 0.8573460000

3-trifluoromethyl-5-aminoisoxazole (Is-3)
C 0.0000000000 0.0000000000 0.0000000000
C -1.4884460000 0.1975960000 0.0025960000
N -1.9896290000 1.4112760000 -0.0015300000
O -3.3848090000 1.2030370000 0.0019220000
C -3.6405710000 -0.1198320000 0.0089010000
C -2.4655280000 -0.8281360000 0.0074170000
H -2.3185020000 -1.8964390000 0.0173520000
N -4.9614340000 -0.4776580000 0.0727210000
H -5.6127200000 0.2332580000 -0.2368180000
H -5.1803100000 -1.4031060000 -0.2704610000
F 0.6689660000 1.1598230000 -0.0182810000
F 0.3873000000 -0.6931720000 1.0931550000
F 0.3775050000 -0.7215610000 -1.0790470000

3-phenyl-2*H*-azirine-2-carboxamide (Az-1)
C 0.0000000000 0.0000000000 0.0000000000
N 0.6468150000 1.3428040000 0.4157210000
C 1.3762350000 0.3159020000 0.3587020000
C 2.7376060000 -0.1392950000 0.5444170000
C 3.0624600000 -1.4872810000 0.3364030000
C 4.3750270000 -1.9199380000 0.5122040000
C 5.3637200000 -1.0107010000 0.8956180000
C 5.0421270000 0.3346130000 1.1055910000
C 3.7336360000 0.7732010000 0.9315500000
H 3.4669440000 1.8134420000 1.0932320000
H 5.8134880000 1.0379330000 1.4062300000
H 6.3868020000 -1.3499810000 1.0333740000
H 4.6269340000 -2.9645520000 0.3535350000
H 2.2818490000 -2.1838620000 0.0448990000
C -0.8925770000 -0.6500840000 1.0196840000
O -0.6031650000 -0.7065870000 2.2056280000
N -2.0315190000 -1.2190530000 0.5019230000
H -2.3809800000 -0.9349860000 -0.4023350000
H -2.7148470000 -1.5442030000 1.1729150000
H -0.2921970000 -0.0744670000 -1.0491690000

H -1.3305280000 -1.1698340000 1.2729270000
H 0.3478860000 -1.7441330000 1.2883270000

3-*tert*-butyl-2*H*-azirine-2-carboxamide (Az-2)
C 0.0000000000 0.0000000000 0.0000000000
C 1.2778830000 -0.2545520000 -0.7339140000
C 2.6223330000 0.2050670000 -1.0790890000
C 3.7843220000 0.0708280000 -0.1385190000
O 4.5991350000 0.9719930000 -0.0007960000
N 3.8262630000 -1.1051810000 0.5764400000
H 3.3515870000 -1.9148090000 0.1973230000
H 4.6903100000 -1.2783620000 1.0731420000
N 1.8469800000 -1.0811520000 -1.4911640000
H 2.7693080000 0.9630480000 -1.8460330000
C 0.3354990000 0.2380660000 1.4892040000
H 1.0080930000 1.0933820000 1.6119410000
H -0.5863640000 0.4442380000 2.0445360000
H 0.8178280000 -0.6381200000 1.9345250000
C -0.6433110000 1.2720740000 -0.5989620000
H -1.5646900000 1.5079800000 -0.0548210000
H 0.0294540000 2.1325980000 -0.5190200000
H -0.8975270000 1.1295120000 -1.6548250000
C -0.9309200000 -1.2137820000 -0.1699550000
H -1.8798550000 -1.0352270000 0.3476220000
H -1.1458750000 -1.4015100000 -1.2271510000
H -0.4786370000 -2.1195520000 0.2476990000

3-trifluoromethyl-2*H*-azirine-2-carboxamide (Az-3)
C 0.0000000000 0.0000000000 0.0000000000
N 0.8264740000 -0.3704480000 -1.2687950000
C 1.3624850000 0.3206290000 -0.3743230000
C 2.6696860000 0.9740700000 -0.0505180000
F 3.5274530000 0.8588390000 -1.0715560000
F 3.2090290000 0.3803630000 1.0326960000
F 2.4916760000 2.2720940000 0.2257940000
C -1.0628590000 1.0487220000 -0.2032460000
O -0.8633090000 2.0352590000 -0.8944380000
N -2.2284240000 0.8189690000 0.4727620000
H -2.4422260000 -0.0830430000 0.8723290000
H -2.9994390000 1.4483720000 0.2956680000
H -0.1991170000 -0.8275700000 0.6805610000

2-phenyl-5-aminooxazole (Ox-1)

N 0.0000000000 0.0000000000 0.0000000000
C 1.0573270000 0.8906520000 0.0741140000
C 1.1874320000 2.2501990000 0.0742060000
N 2.5361300000 2.5700800000 0.0613210000
C 3.1649140000 1.4333400000 0.0614250000
O 2.3061630000 0.3468200000 0.0645350000
C 4.5983680000 1.1761460000 0.0677630000
C 5.1098110000 -0.1317660000 0.0578440000
C 6.4868900000 -0.3452410000 0.0640740000
C 7.3686790000 0.7370510000 0.0793260000
C 6.8631340000 2.0407560000 0.0883870000
C 5.4899980000 2.2631760000 0.0831010000
H 5.0866380000 3.2705430000 0.0901920000
H 7.5436420000 2.8880620000 0.0999990000
H 8.4419480000 0.5673130000 0.0834710000
H 6.8716770000 -1.3616160000 0.0558910000
H 4.4262700000 -0.9744690000 0.0435900000
H 0.4160490000 3.0064190000 0.0813380000
H -0.8882490000 0.4226120000 0.2427050000
H 0.1532070000 -0.8531200000 0.5283720000

2-*tert*-butyl-5-aminooxazole (Ox-2)
C 0.0000000000 0.0000000000 0.0000000000
N 1.9704870000 1.6018120000 0.0031270000
C 3.3508970000 1.3990370000 0.0131470000
C 3.5964790000 0.0603850000 -0.0019540000
O 2.3954900000 -0.5896580000 -0.0163580000
C 1.4467600000 0.4202300000 -0.0070180000
N 4.7264710000 -0.7417010000 -0.0921100000
H 5.5769100000 -0.2322390000 0.1191690000
H 4.6647790000 -1.5801930000 0.4779230000
H 4.0518240000 2.2208760000 0.0282110000
C -0.8821840000 1.2599860000 0.0088230000
H -0.6859560000 1.8735150000 0.8931150000
H -0.6930410000 1.8801880000 -0.8723070000
H -1.9391320000 0.9698130000 0.0118060000
C -0.2964930000 -0.8422210000 -1.2630340000
H -0.1197090000 -0.2585400000 -2.1732970000
H 0.3356860000 -1.7352030000 -1.3032210000
H -1.3445050000 -1.1645400000 -1.2628780000
C -0.2821830000 -0.8489710000 1.2619170000
H -0.0937980000 -0.2710380000 2.1737140000
H -1.3305280000 -1.1698340000 1.2729270000

H 0.3478860000 -1.7441330000 1.2883270000

2-trifluoromethyl-5-aminooxazole (Ox-3)
C 0.0000000000 0.0000000000 0.0000000000
C -1.4383330000 0.4043150000 -0.0247900000
O -2.3626620000 -0.6119550000 -0.0258580000
C -3.5628680000 0.0327940000 -0.0019810000
C -3.3213870000 1.3802650000 0.0135300000
N -1.9498970000 1.5875910000 -0.0023620000
H -4.0252340000 2.1987180000 0.0316840000
N -4.6854080000 -0.7625040000 -0.0777300000
H -4.5822540000 -1.6700430000 0.3626670000
H -5.5368980000 -0.2964880000 0.2098670000
F 0.2882360000 -0.8452730000 -1.0133120000
F 0.3096730000 -0.6467270000 1.1473030000
F 0.7894840000 1.0744770000 -0.1029250000

Excitation energies, oscillator strengths, and orbital character for transitions

Table S1. Excitation energies, oscillator strengths, and orbital character for calculated transitions of isomer trio **1** (phenyl-substituted) with an oscillator strength of 0.1 or higher.

Excited State #	Energy (eV)	Wavelength (nm)	Osc. Strength	Orbital character and amplitudes
**Is-1**				
3	4.9586	250.04	0.2411	40 -> 43 -0.38717<br>41 -> 43 0.46894<br>41 -> 44 -0.26475<br>42 -> 44 -0.11405
4	5.1352	241.44	0.2989	40 -> 43 0.12418<br>41 -> 43 0.27234<br>41 -> 44 0.16661<br>42 -> 44 0.59087
6	5.8749	211.04	0.2034	40 -> 43 -0.33476<br>40 -> 47 0.11690<br>41 -> 44 0.50142<br>42 -> 47 0.15251
8	6.1029	203.16	0.2941	39 -> 43 -0.13467<br>42 -> 46 -0.11218<br>42 -> 47 0.60616
**Az-1**				
1	4.6255	268.04	0.1812	39 -> 43 0.15756<br>40 -> 43 0.31994<br>42 -> 43 0.57453
4	5.0636	244.85	0.3365	39 -> 43 0.17681

				40 -> 43 0.55341<br>41 -> 44 0.11570<br>42 -> 43 -0.29405
7	5.9034	210.02	0.1294	40 -> 44 0.11361<br>41 -> 43 -0.19639<br>41 -> 45 0.10103<br>41 -> 48 0.12987<br>42 -> 44 0.60442
9	6.2836	197.31	0.1165	37 -> 43 0.11852<br>40 -> 44 0.53089<br>41 -> 44 -0.24612<br>41 -> 45 -0.30015
10	6.3304	195.85	0.3583	37 -> 43 -0.30465<br>38 -> 44 0.11465<br>39 -> 44 -0.11940<br>39 -> 45 -0.11684<br>40 -> 44 0.21790<br>41 -> 44 0.45800<br>41 -> 45 -0.19923
**Ox-1**				
1	3.7755	328.39	0.5771	42 -> 43 0.65205

Table S2. Excitation energies, oscillator strengths, and orbital character for calculated transitions of isomer trio **2** (*tert*-butyl-substituted) with an oscillator strength of 0.1 or higher.

Excited State #	Energy (eV)	Wavelength (nm)	Osc. Strength	Orbital character and amplitudes
**Is-2**				
2	5.581	222.15	0.287	38 -> 39 0.64176
**Az-2**				
5	6.5916	188.09	0.2087	37 -> 39 0.12017<br>37 -> 40 0.62012<br>38 -> 41 -0.18237
7	6.9333	178.82	0.2366	36 -> 40 0.56843<br>36 -> 41 -0.16269<br>37 -> 40 -0.11361<br>38 -> 40 0.13149<br>38 -> 41 -0.13717<br>38 -> 43 0.10899
8	7.0735	175.28	0.1017	34 -> 39 0.10364<br>35 -> 39 -0.43681<br>37 -> 41 0.48832
**Ox-2**				
2	5.2076	238.08	0.2893	38 -> 40 0.65368

Table S3. Excitation energies, oscillator strengths, and orbital character for calculated transitions of isomer trio **3** (trifluoromethyl-substituted) with an oscillator strength of 0.1 or higher.

Excited State #	Energy (eV)	Wavelength (nm)	Osc. Strength	Orbital character and amplitudes
**Is-3**				
1	5.1850	239.12	0.2022	38 -> 39 0.64621
4	6.5759	188.54	0.1179	37 -> 39 -0.15193<br>38 -> 42 0.64189<br>38 -> 44 0.15784
10	7.4301	166.87	0.1546	35 -> 39 -0.16609<br>37 -> 39 0.55142<br>38 -> 42 0.14780<br>38 -> 44 -0.19409<br>38 -> 47 -0.17018
**Az-3**				
5	6.7126	184.70	0.1576	37 -> 40 0.59262<br>38 -> 40 -0.20840<br>38 -> 41 -0.13900
7	7.0795	175.13	0.2769	35 -> 39 0.34396<br>36 -> 40 0.55131<br>37 -> 41 -0.12243
9	7.2557	170.88	0.1035	34 -> 40 0.12428<br>35 -> 39 0.51424<br>36 -> 40 -0.33693<br>36 -> 41 0.12199
**Ox-3**				
1	4.8572	255.26	0.2826	38 -> 39 0.64708
4	6.2820	197.36	0.1219	38 -> 42 0.65186<br>38 -> 43 -0.11128<br>38 -> 46 -0.10166<br>38 -> 48 -0.12360

Comparison of extra diffuse basis set

To confirm that the potential charge transfer nature of the transitions involved were accurately captured at the level of theory, we revisited the CF_3 isomer trio with the more diffuse 6-311++G(2d,p) basis set. The resulting simulated absorption spectra are comparable to those reported above.

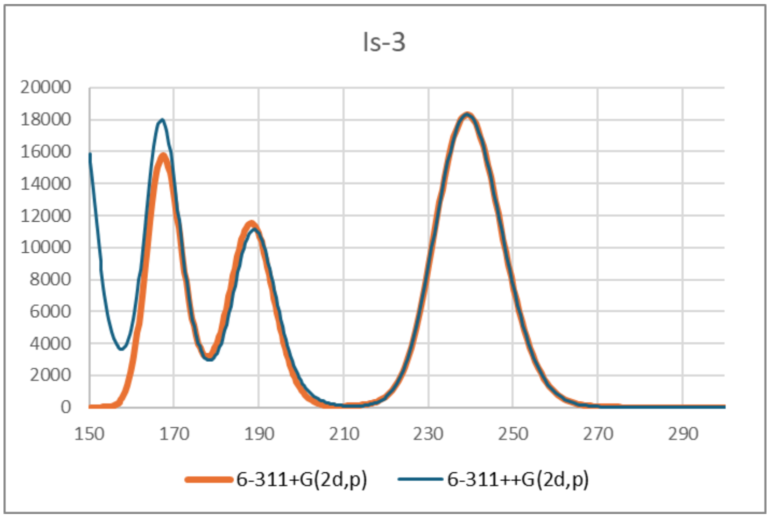

Figure S17. Comparison of simulated absorption spectra for **Is-3** at the B3LYP/6-311+G(2d,p) and B3LYP/6-311++G(2d,p) levels of theory.

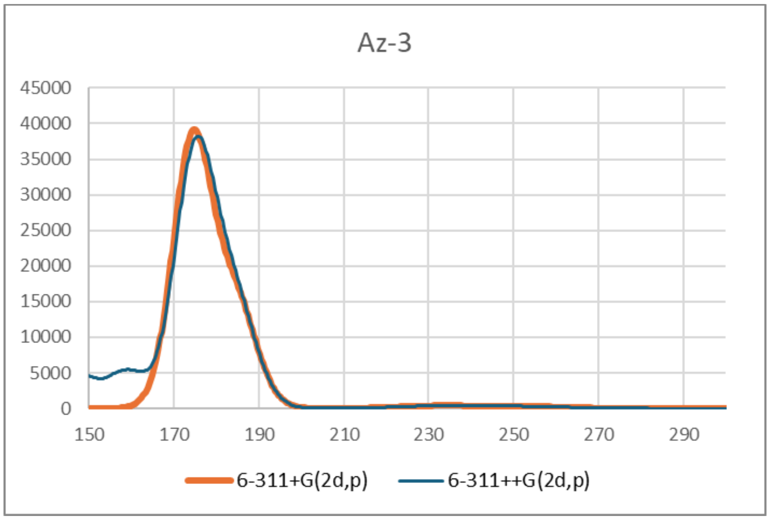

Figure S18. Comparison of simulated absorption spectra for **Az-3** at the B3LYP/6-311+G(2d,p) and B3LYP/6-311++G(2d,p) levels of theory.

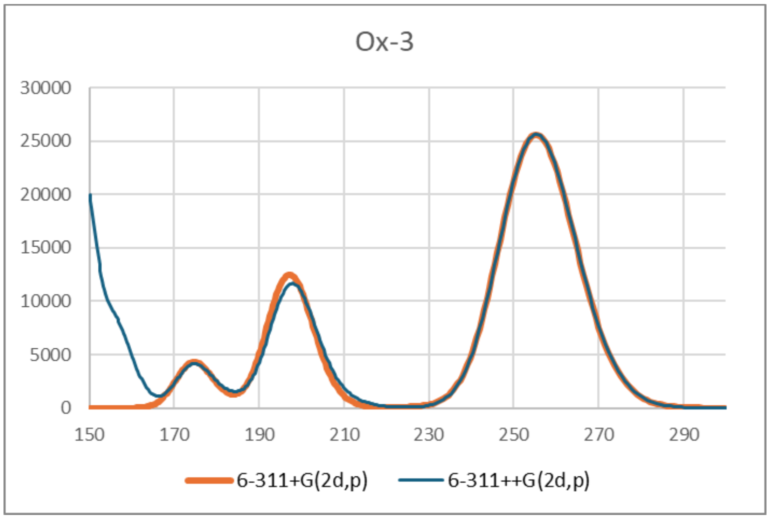

Figure S19. Comparison of simulated absorption spectra for **Ox-3** at the B3LYP/6-311+G(2d,p) and B3LYP/6-311++G(2d,p) levels of theory.

Relative energies of simulated species

Table S4. Relative free energy calculated at the B3LYP/6-311+G(2d,p) level of theory.

	isoxazole	2*H*-azirine	oxazole
R^1	Relative free energy (kcal/mol)		
Ph	0	-3.26	-22.46
t-Bu	0	-5.46	-21.16
CF_3	0	+4.80	-18.49

Population analysis

To better understand the electronic differences underpinning the difference in behavior between isomer trios 2 and 3 with isomer trio 1, we present the computed charge densities of the core and substituents for the three isomer trios. Populations were calculated through two methods, Mulliken and Atomic Polar Tensor. There are differences in the resulting populations which we are unable to reconcile at this moment, but both analyses are presented below for transparency.

Mulliken analysis of the charge density of each species corroborates the orbital arguments from the main text outlining the similarities in isomer trio 2 and 3 in contrast with isomer trio 1. The three compounds were divided into the "core" (isoxazole, carbonyl-2*H*-azirine, or oxazole), amine group, and R1 substituent and the overall charge of each fragment in each of the three species of each isomer trio is presented below. For ease of comparison, the change in electron density for a fragment from isoxazole to carbonyl-2*H*-azirine is also added as a column (indicated as Δ). Isomer trios 2 and 3 show a very slight electron migration from the core and amine to their respective R^1 substituent in transforming from isoxazole to carbonyl-2*H*-azirine. In contrast, the phenyl-substituted isomer trio 1 shows a pronounced transfer of charge from the phenyl substituent to the core when Is-1 is converted to Az-1.

In contrast, Atomic Polar Tensor (APT) populations demonstrate that the charge density does not change much between the three isomers of any particular trio, and the three trios show fairly consistent behavior of a negatively charged amine substituent and a positive core. These results do not follow the orbital arguments outlined in the main text, indicating that any movement of charge is temporary (perhaps only existing as long as the formation of the nitrene intermediate).

Table S5. Population analysis of charge density on a per-fragment basis

Ph-NH2				
	Azirine	Isox	Ox	Δ (Is to Az)
Core	-0.26	0.36	0.25	-0.62
Amine	0.04	0.00	-0.02	0.05
R1	0.22	-0.36	-0.22	0.57
tbut-NH2				
	Azirine	Isox	Ox	
Core	-0.29	-0.32	-0.24	0.04
Amine	0.04	0.01	-0.03	0.03
R1	0.25	0.32	0.27	-0.07
CF3-NH2				
	Azirine	Isox	Ox	
Core	-0.14	-0.26	-0.20	0.12
Amine	0.05	0.04	0.00	0.01
R1	0.09	0.22	0.21	-0.13

REFERENCES

(1) Boston, E. *265nm UV LED Catalog*. www.boselec.com. https://www.boselec.com/wp-content/uploads/Linear/Violumas/ViolumasLiterature/255-nm-UVLED-Catalog-BEC-05-12-25.pdf (accessed 2025-11-20).

(2) Frisch, M. J.; Trucks, G. W.; Schlegel, H. B.; Scuseria, G. E.; Robb, M. A.; Cheeseman, J. R.; Montgomery, J. A., Jr.; Vreven, T.; Kudin, K. N.; Burant, J. C.; Millam, J. M.; Iyengar, S. S.; Tomasi, J.; Barone, V.; Mennucci, B.; Cossi, M.; Scalmani, G.; Rega, N.; Petersson, G. A.; Nakatsuji, H.; Hada, M.; Ehara, M.; Toyota, K.; Fukuda, R.; Hasegawa, J.; Ishida, M.; Nakajima, T.; Honda, Y.; Kitao, O.; Nakai, H.; Klene, M.; Li, X.; Knox, J. E. Gaussian 03, Revision C.02.

(3) Lee, C.; Yang, W.; Parr, R. G. Development of the Colle-Salvetti Correlation-Energy Formula into a Functional of the Electron Density. *Phys. Rev. B* **1988**, *37* (2), 785–789. https://doi.org/10.1103/PhysRevB.37.785.

(4) Vosko, S. H.; Wilk, L.; Nusair, M. Accurate Spin-Dependent Electron Liquid Correlation Energies for Local Spin Density Calculations: A Critical Analysis. *Can. J. Phys.* **1980**, *58* (8), 1200–1211. https://doi.org/10.1139/p80-159.

(5) Stephens, P. J.; Devlin, F. J.; Chabalowski, C. F.; Frisch, M. J. Ab Initio Calculation of Vibrational Absorption and Circular Dichroism Spectra Using Density Functional Force Fields. *J. Phys. Chem.* **1994**, *98* (45), 11623–11627. https://doi.org/10.1021/j100096a001.

(6) Becke, A. D. Density-functional Thermochemistry. III. The Role of Exact Exchange. *J. Chem. Phys.* **1993**, *98* (7), 5648–5652. https://doi.org/10.1063/1.464913.

(7) Hehre, W. J.; Ditchfield, R.; Pople, J. A. Self—Consistent Molecular Orbital Methods. XII. Further Extensions of Gaussian—Type Basis Sets for Use in Molecular Orbital Studies of Organic Molecules. *J. Chem. Phys.* **1972**, *56* (5), 2257–2261. https://doi.org/10.1063/1.1677527.

(8) Hariharan, P. C.; Pople, J. A. The Influence of Polarization Functions on Molecular Orbital Hydrogenation Energies. *Theor. Chim. Acta* **1973**, *28* (3), 213–222. https://doi.org/10.1007/BF00533485.

(9) Krishnan, R.; Binkley, J. S.; Seeger, R.; Pople, J. A. Self-consistent Molecular Orbital Methods. XX. A Basis Set for Correlated Wave Functions. *J. Chem. Phys.* **1980**, *72* (1), 650–654. https://doi.org/10.1063/1.438955.

(10) Tomasi, J.; Mennucci, B.; Cammi, R. Quantum Mechanical Continuum Solvation Models. *Chem. Rev.* **2005**, *105* (8), 2999–3094. https://doi.org/10.1021/cr9904009.

(11) Avogadro: An Open-Source Molecular Builder and Visualization Tool. http://avogadro.cc/.

(12) Hanwell, M. D.; Curtis, D. E.; Lonie, D. C.; Vandermeersch, T.; Zurek, E.; Hutchison, G. R. Avogadro: An Advanced Semantic Chemical Editor, Visualization, and Analysis Platform. *J. Cheminformatics* **2012**, *4* (1), 17. https://doi.org/10.1186/1758-2946-4-17.

(13) Liu, H.; Yan, Y.; Li, M.; Zhang, X. An Enantioselective Aza-Friedel–Crafts Reaction of 5-Aminoisoxazoles with Isatin-Derived N-Boc Ketimines. *Org. Biomol. Chem.* **2021**, *19* (17), 3820–3824. https://doi.org/10.1039/D1OB00374G.

(14) Rowbottom, M. W.; Faraoni, R.; Chao, Q.; Campbell, B. T.; Lai, A. G.; Setti, E.; Ezawa, M.; Sprankle, K. G.; Abraham, S.; Tran, L.; Struss, B.; Gibney, M.; Armstrong, R. C.; Gunawardane, R. N.; Nepomuceno, R. R.; Valenta, I.; Hua, H.; Gardner, M. F.; Cramer, M. D.; Gitnick, D.; Insko, D. E.; Apuy, J. L.; Jones-Bolin, S.; Ghose, A. K.; Herbertz, T.; Ator, M. A.; Dorsey, B. D.; Ruggeri, B.; Williams, M.; Bhagwat, S.; James, J.; Holladay, M. W. Identification of 1-(3-(6,7-Dimethoxyquinazolin-4-Yloxy)Phenyl)-3-(5-(1,1,1-Trifluoro-2-Methylpropan-2-Yl)Isoxazol-3-Yl)Urea Hydrochloride (CEP-32496), a Highly Potent and Orally Efficacious Inhibitor of V-RAF Murine Sarcoma Viral Oncogene Homologue B1 (BRAF) V600E. *J. Med. Chem.* **2012**, *55* (3), 1082–1105. https://doi.org/10.1021/jm2009925.

(15) Ge, Y.; Sun, W.; Pei, B.; Ding, J.; Jiang, Y.; Loh, T.-P. Hoveyda–Grubbs II Catalyst: A Useful Catalyst for One-Pot Visible-Light-Promoted Ring Contraction and Olefin Metathesis Reactions. *Org. Lett.* **2018**, *20* (9), 2774–2777. https://doi.org/10.1021/acs.orglett.8b00971.
